# Chronic Parasitic Infection Maintains High Frequencies of Short-Lived Ly6C^+^CD4^+^ Effector T Cells That Are Required for Protection against Re-infection

**DOI:** 10.1371/journal.ppat.1004538

**Published:** 2014-12-04

**Authors:** Nathan C. Peters, Antonio J. Pagán, Phillip G. Lawyer, Timothy W. Hand, Eric Henrique Roma, Lisa W. Stamper, Audrey Romano, David L. Sacks

**Affiliations:** 1 Intracellular Parasite Biology Section, Laboratory of Parasitic Diseases, National Institute of Allergy and Infectious Diseases, National Institutes of Health, Bethesda, Maryland, United States of America; 2 Department of Microbiology, Center for Immunology, University of Minnesota Medical School, Minneapolis, Minnesota, United States of America; University of Massachusetts Medical School, United States of America

## Abstract

In contrast to the ability of long-lived CD8^+^ memory T cells to mediate protection against systemic viral infections, the relationship between CD4^+^ T cell memory and acquired resistance against infectious pathogens remains poorly defined. This is especially true for T helper 1 (Th1) concomitant immunity, in which protection against reinfection coincides with a persisting primary infection. In these situations, pre-existing effector CD4 T cells generated by ongoing chronic infection, not memory cells, may be essential for protection against reinfection. We present a systematic study of the tissue homing properties, functionality, and life span of subsets of memory and effector CD4 T cells activated in the setting of chronic *Leishmania major* infection in resistant C57Bl/6 mice. We found that pre-existing, CD44^+^CD62L^−^T-bet^+^Ly6C^+^ effector (T_EFF_) cells that are short-lived in the absence of infection and are not derived from memory cells reactivated by secondary challenge, mediate concomitant immunity. Upon adoptive transfer and challenge, non-dividing Ly6C^+^ T_EFF_ cells preferentially homed to the skin, released IFN-γ, and conferred protection as compared to CD44^+^CD62L^−^Ly6C^−^ effector memory or CD44^+^CD62L^+^Ly6C^−^ central memory cells. During chronic infection, Ly6C^+^ T_EFF_ cells were maintained at high frequencies via reactivation of T_CM_ and the T_EFF_ themselves. The lack of effective vaccines for many chronic diseases may be because protection against infectious challenge requires the maintenance of pre-existing T_EFF_ cells, and is therefore not amenable to conventional, memory inducing, vaccination strategies.

## Author Summary

Naturally acquired resistance to reinfection by numerous infectious pathogens including *Leishmania*, *Plasmodium*, *Mycobacterium*, and parasitic worms, typically coincides with an ongoing primary infection. This natural resistance to reinfection, termed concomitant immunity, is often referred to as a memory response and provides the rationale for the vaccine effort against these infectious pathogens. However, immune memory is mediated by populations of long-lived cells that do not require an ongoing primary infection to mediate protection. The requirement for chronic infection to maintain concomitant immunity suggests that the critical cells that mediate this immunity are not memory cells. In the present study we define short-lived effector T cells that pre-exist secondary challenge, not memory cells, as the critical cells that mediate concomitant immunity. These observations provide direct evidence on a cellular level that conventional vaccination strategies against chronic infectious diseases, whose development is predicated upon the belief that concomitant immunity can be mediated by long-lived memory cells, are unlikely to succeed.

## Introduction

Chronic infectious diseases, including those caused by *Leishmania*, *Plasmodium*, *Mycobacterium*, and parasitic worms, continue to be a major cause of morbidity and mortality in the world. Understanding the critical factors mediating protective immunity to these diseases and whether this immunity can be mediated by memory cells is likely to contribute to a long-term solution, including the feasibility of developing a vaccine. Observations employing infection with lymphphocytic choriomeningitis virus (LCMV) and *Listeria monocytogenes* have established a paradigm of CD8^+^ T cell memory in which stable populations of central (T_CM_) and effector (T_EM_) memory cells are found after clearance of a primary infection. These CD8^+^ memory cells mediate protective immunity upon secondary infection [1], [2]. In contrast, the nature of CD4^+^ T cell memory is less clear. This is especially true in cases of CD4-mediated concomitant immunity, where protection against reinfection coincides with the persistence of a primary infection. Infections in which concomitant immunity is thought to play a significant role include Malaria, Leishmaniasis, Tuberculosis, some forms of Salmonellosis, and helminthic infections [3]–[10]. The immune response against re-infection in these settings is often referred to as a memory response, which, as most commonly defined, is constituted by a population of long-lived cells that do not require the continued presence of antigen. However, chronic infection may provide a persistent source of antigen that sustains effector CD4+ T cells. Pre-existing and infection dependent effector cells may be critical to provide protection upon re-infection [6]–[8], [11].

In a murine model of cutaneous leishmaniasis, Zaph et al. [12] demonstrated that CD4^+^CD62L^+^ T cells with the functional attributes of T_CM_ cells were maintained in the absence of persistent *Leishmania* parasites and could mediate delayed protection to re-challenge by needle inoculation. The role of memory cells in *Leishmania* concomitant immunity has been questioned, however, by studies suggesting that immunity is either completely lost or is suboptimal when the chronic primary infection is eliminated [12]–[15]. Similar observations exist in cancer and malaria [16], [17]. We have recently reported that the clearest correlate of effective concomitant immunity against natural transmission of *Leishmania* by the bite of an infected sand fly is the rapid recruitment (within 24 hours) of IFN-γ-producing CD4^+^ T cells to the cutaneous bite site. In contrast, non-living vaccines mediate delayed immunity to needle challenge similar to that reported for T_CM_ cells, and provide no protection to infected sand fly challenge [11], [18]. The rapid CD4^+^ T cell response is necessary to counteract the disease exacerbating inflammatory response elicited by natural sand fly transmission [11]. The nature of the rapidly responding CD4^+^ T cells that mediate concomitant immunity, including their frequency and tissue distribution during the course of chronic infection, their migration to and function within the challenge site, and most critically, their life span and memory potential, have not been determined. Here, we define those cells that mediate concomitant immunity as pre-existing, short-lived, T-bet^hi^Ly6C^+^ T_EFF_ cells that, despite their short life span in the absence of infection, are maintained as the dominant population of antigen experienced cells throughout chronic infection.

## Results

### Analysis of concomitant immunity in cutaneous Leishmaniasis

**Figure 1:**
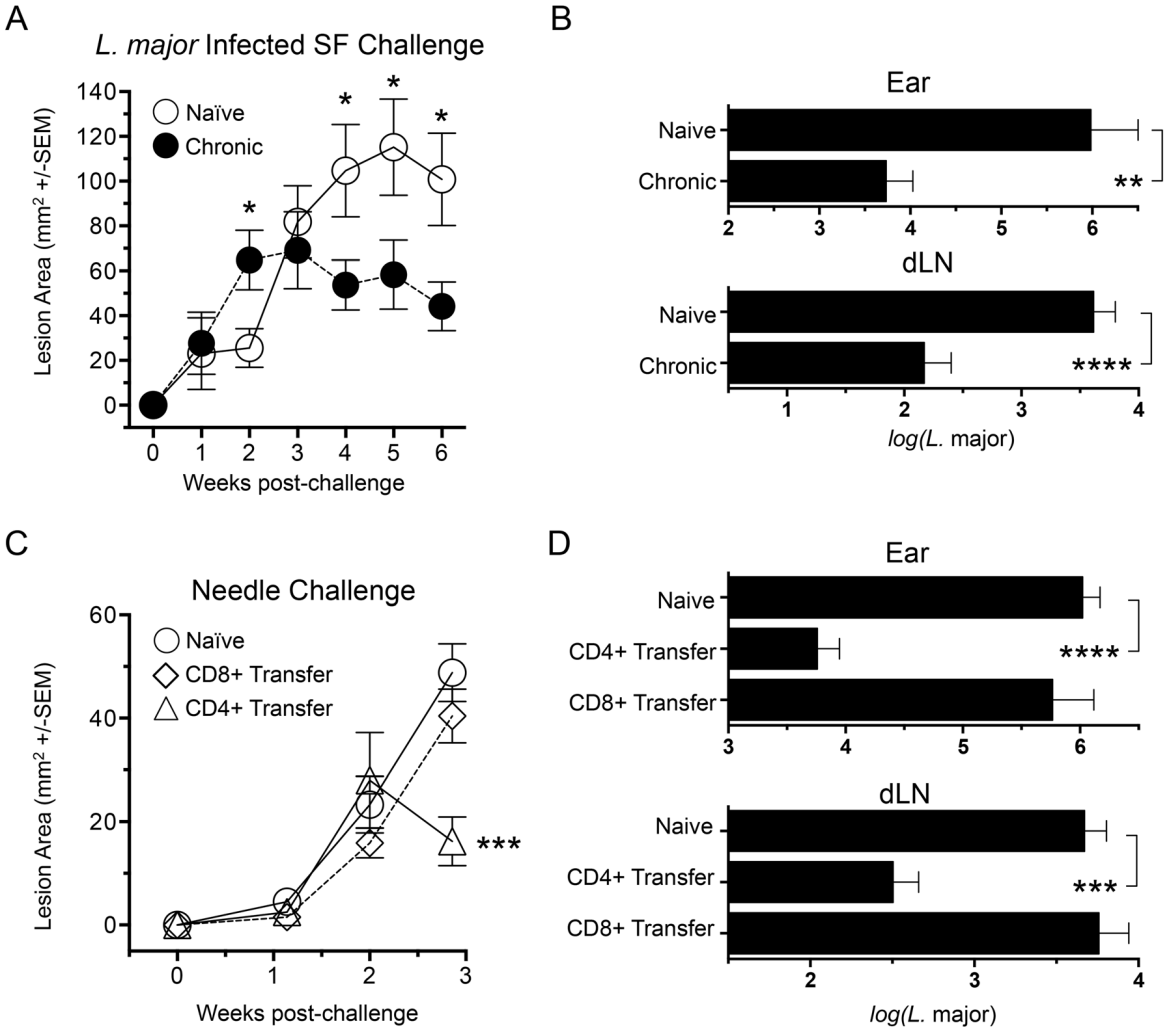
Chronic primary *L. major* infection protects against cutaneous Leishmaniasis initiated by infected sand fly challenge; CD4^+^ Th cells transfer protection. (**A and B**) Ears of naïve, age matched control mice (Naïve), or mice with a healed but chronic primary infection (Chronic) were exposed to the bites of 6 *L. major*-infected *P. duboscqi* sand flies (*L.m.* Infected SF Challenge, n = 12–16 ears per group). (**C and D**) Polyclonal TCRβ^+^CD4^+^CD8^−^CD11b^−^NK1.1^−^MHCII^−^ (CD4^+^) or TCRβ^+^CD4^−^CD8^+^CD11b^−^NK1.1^−^MHCII^−^ (CD8^+^) T cells were FACS sorted from 15 chronic mice and independently transferred into 3 wild type naïve recipient mice at their pre-transfer physiological ratios (approximately 1×10^7^ CD4 cells or 8×10^6^ CD8 T cells). One day following transfer, ears were challenged with 5000 *L. major* metacyclic promastigotes, a dose meant to approximate the number of parasites transmitted by 6 *L. major*-infected *P. duboscqi* sand flies (Naïve control group n = 8, Transfer groups n = 6 ears per group). (**A and C**) Ear lesions were measured weekly in two dimensions and the lesion area determined. **In A:** (*) p<0.05, Naïve versus chronic; in B: (***) p = 0.0007 versus Naive. (**B and D**) Parasite loads in individual ears or dLNs at six (B) or three (D) weeks post-challenge. (**) p = 0.0011, (***) p = 0.0003, (****) p<0.0001.

People with a healed but chronic primary *Leishmania* infection are highly resistant to reinfection following natural exposure to infected sand flies [19]. The same is true in an experimental setting where mice with a healed but chronic primary infection in the footpad presented with significantly smaller lesions compared to naïve mice starting at 4 weeks post-exposure of the ear dermis to infected sand fly bites (p≤0.04) (Fig. 1A). Chronic mice also had significantly fewer parasites in the sand fly challenged ear (178-fold reduction, p = 0.0011) and ear dLN (27-fold reduction, p≤0.0001) at 6 weeks post-infection (Fig. 1B). This is in contrast to non-living vaccines, which do not protect against sand fly transmitted disease either in mice or humans [11], [18], [20].

While CD8 cells have been shown to play a role in secondary immune responses to *L. major* infection [21], protection conferred following adoptive transfer of bulk immune cells versus CD4^+^ T cells alone has shown that CD4^+^ T cells alone are sufficient [12]. We expanded upon these observations by determining the protective capacity of CD4^+^ or CD8^+^ T cells from chronically infected mice following adoptive transfer into naïve recipients at their pre-existing physiological ratios (Figure 1, C and D). Because of the constraints on the numbers of purified cells available for transfer, in conjunction with the enormous variability of successful transmission by sand fly bite, needle challenge was employed to insure that an infection outcome could be followed in the relatively small numbers of mice per group that we are able to employ in our adoptive transfer system. We measured early control of parasite numbers following needle challenge as this has consistently provided the best correlate of protective immunity against infected sand fly challenge, and protection at later time points against needle challenge does not correlate with protection against sand fly challenge [11], [18]. We found that CD4^+^ Th cells conferred protection versus control animals at 21 days post-challenge, significantly reducing lesion size (p = 0.0007) (Fig. 1C) and parasite loads in the ear and ear dLN (p≤0.0002) (Fig. 1 D). In contrast, CD8^+^ T cells conferred no protection versus naïve controls. Subsequent studies were designed to determine the nature of the CD4^+^ Th cells that confer this protection.

**Figure 2:**
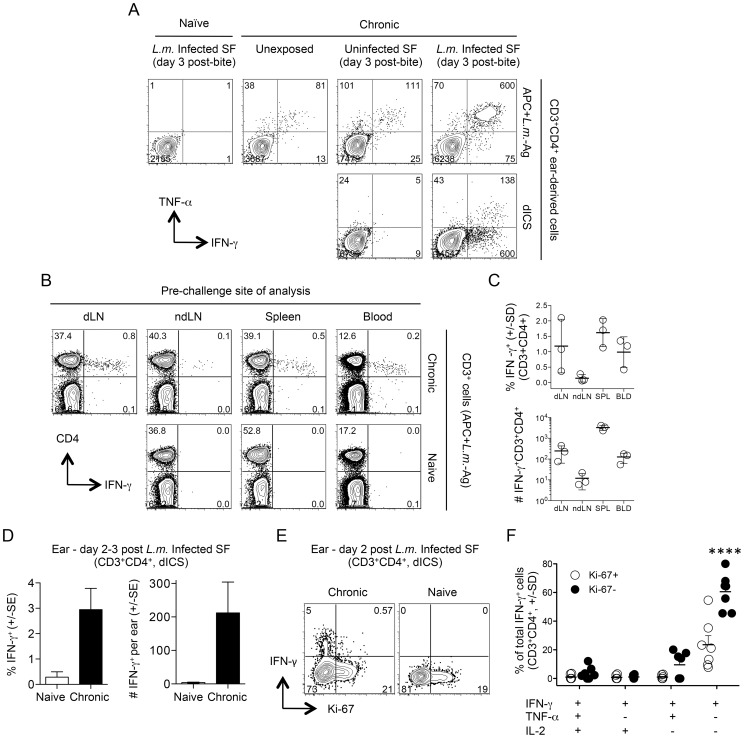
Analysis of the early concomitant immune response following exposure to the bites of *L. major*-infected sand flies. (**A, D–F**) Ears of naïve mice (Naïve) or uninfected ears of mice with a chronic primary infection in the left hind footpad (Chronic) were unexposed, or exposed to the bites of uninfected sand flies (Uninfected SF) or *L. major* infected sand flies (*L.m.* Infected SF). 2–3 days later CD3^+^CD4^+^ from single ears were analyzed by flow cytometry. (**A**) Representative intracellular staining for cytokine-producing cells after over-night (14 hours) *in-vitro* stimulation with APC+*L.m.*-Ag (top panels) or direct intracellular staining (dICS) (bottom panels) on day 3 post-challenge. Numbers indicate the number of cells per ear in each quadrant. (**B and C**) Cells from the indicated locations were isolated from perfused naive or chronic mice and stimulated with APC+*L.m.*-Ag. (**B**) Representative dot-plots of an individual naïve or chronic mouse. Numbers represent the frequency of cells in each quadrant. (**C**) Percentage and relative # (per site or ml of blood) of IFN-γ^+^ cells from n = 3 individual chronic mice. (**D**) Percentage or number of IFN-γ^+^ cells per ear following exposure to infected sand fly bites and dICS. Results are pooled from 3 independent experiments, n = 10 (naïve) or 11 (chronic) ears. (**E**) Representative dot-plot of IFN-γ and Ki-67 expression following dICS. Numbers in dot plots represent frequency. (**F**) Cytokine-production as a function of Ki-67 expression by IFN-γ^+^CD3^+^CD4^+^ ear cells following dICS (n = 7 ears). ****, p≤0.0001 versus all other groups. Data is representative of 3 similar experiments.

The rapid recruitment (within 3 days) of IFN-γ^+^CD4^+^ Th cells to the cutaneous site of infected sand fly bite in mice with a chronic *L. major* infection is the strongest correlate of protection against natural re-infection [11]. We exposed the ear dermis of mice with a healed chronic infection in the footpad to the bites of *L.m.*-infected flies and 3 days later dermal derived CD3^+^CD4^+^ T cells were analyzed for cytokine production. Following antigen re-stimulation, large numbers of cells produced IFN-γ and TNF-α (Fig. 2A, top, right panel). Exposure of chronic mice to the bites of uninfected sand flies also resulted in greater numbers of cytokine positive cells versus unexposed chronic mice, which contained small numbers of pre-existing CD4^+^IFN-γ^+^ cells (Fig. 2A, top, middle panels) [22]. These cells are likely recruited to the skin in an antigen non-specific manner [23] from the blood where significant numbers of T cells with the capacity to make IFN-γ exist before challenge (Fig. 2B and C). Cells recovered from ears exposed to infected sand fly bites and evaluated for cytokine production by direct intracellular staining (dICS, see Figure S1) without antigen or pharmacological re-stimulation contained large numbers of IFN-γ^+^ cells (Fig. 2A, bottom right panel and 2D). In contrast, exposure to the bites of uninfected flies revealed virtually no IFN-γ^+^ cells following dICS, likely due to the absence of antigen (Fig. 2A, bottom left panel). Direct ICS is a highly physiological assessment of antigen-specific cells making cytokine in response to *in-vivo* infection. On day 2 post-infected sand fly exposure, IFN-γ^+^ cells were highly enriched for Ki-67^−^ cells (Fig. 2E), indicating that these cells had not undergone proliferation in the time since challenge and were therefore unlikely to be derived from memory cells reactivated by secondary antigen exposure [12], [24]. Assessment of IFN-γ, TNF-α and IL-2 production by dICS revealed that IFN-γ^+^CD4^+^ T cells were predominantly Ki-67^−^IFN-γ single producers (Fig. 2F), in striking contrast to the predominant IFN-γ^+^TNF-α^+^ phenotype detected following overnight antigen re-stimulation (Fig. 2A).

### Rapidly recruited *L. major*-specific cells are not derived from central memory cells reactivated by secondary challenge

**Figure 3:**
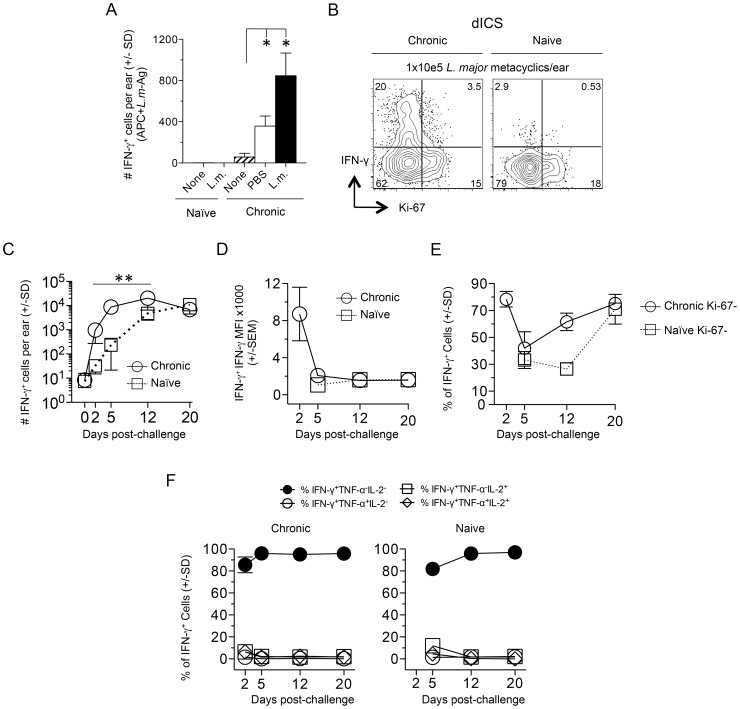
Kinetic analysis of CD4^+^ T cell cytokine production and proliferation history following needle challenge with *L. major*. Ears of naïve or chronic mice were not challenged (None) or challenged by needle inoculation of PBS or 1–2×10^5^ *L.m.* metacyclic promastigotes (*L.m.*). At the indicated time-points post-challenge CD3^+^CD4^+^ T cells from the ear were analyzed by flow-cytometry. (**A**) 20 hours post-inoculation, ear-derived cells were analyzed for IFN-γ-producing CD4^+^ T cells after over-night (14 hours) *in-vitro* APC+*L.m.*-Ag re-stimulation. (*) p<0.05, n = 3. (**B**) Representative dot-plot of IFN-γ and Ki-67 expression following dICS on day 2 post-challenge. (**C**) The number of IFN-γ^+^CD4^+^ T cells per ear following dICS as a function of time p.i. **, p≤0.002 on days 2, 5, and 12 comparing chronic versus naive. (**D**) Average IFN-γ MFI of IFN-γ^+^ cells. (**E**) Percentage of IFN-γ^+^ cells that are Ki-67^−^. (**F**) Kinetic analysis of multi-cytokine producing cells as determined by dICS. Data in (C, E, and F) is pooled from two independent experiments; n = 3 (day 0) or n = 8 (days 2–20) for the Naïve group and n = 7 (day 0) or 6–8 (days 2–20) for the chronic group. Data in (A and B) are representative of two independent experiments.

We extended our findings employing needle challenge, which is far more reproducible in terms of inoculum dose and tissue damage. Twenty hours following needle inoculation with PBS, the number of IFN-γ^+^CD4^+^ T cells following Ag stimulation was significantly greater in chronic mice versus naïve or non-injected chronic controls (Fig. 3A), again demonstrating the recruitment of these cells to the skin in an antigen non-specific manner, likely from the blood. Employing dICS, rapidly recruited IFN-γ^+^ cells observed at day 2 post-needle challenge were also highly enriched for Ki-67^−^ cells (Compare Fig. 2E to Fig. 3B). Kinetic analysis of the secondary response by dICS revealed a marked increase in the relative number of CD4^+^ T cells producing IFN-γ *in-vivo* by day 2 post challenge in chronic versus naïve mice (p≤0.002) (Fig. 3C). Rapidly recruited IFN-γ^+^ cells observed at day 2 expressed high levels of IFN-γ relative to later time points (p<0.002) (Fig. 3D) and were highly enriched for Ki-67^−^, IFN-γ single-producing cells (Fig. 3, E and F).

**Figure 4:**
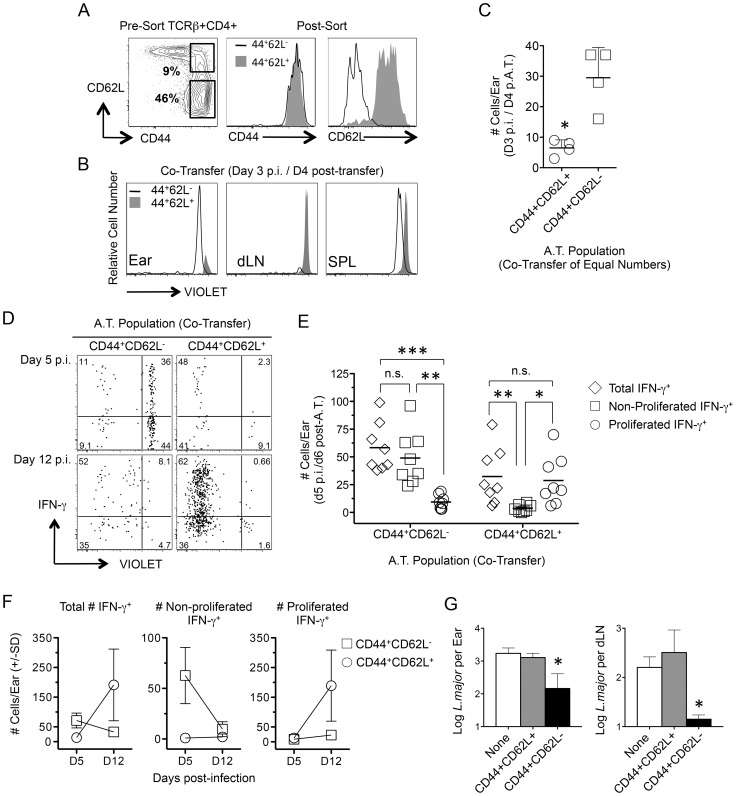
Rapidly recruited antigen-specific cells confer protection and are not derived from central memory cells stimulated by challenge. (**A–F**) CD4^+^CD44^+^CD62L^+^ (T_CM_) and CD4^+^CD44^+^CD62L^−^ T cells were FACS sorted from chronic congenic mice, labeled with VIOLET proliferation dye and co-transferred into naïve UB-gfp mice (see Fig. S2). One day post-transfer recipient mice were challenged with *L.m*. Ears or purified CD4^+^ T cells from the spleen or dLNs of recipient mice were analyzed at the indicated time-points post-challenge by flow cytometry (histograms and dot-plots gated on TCRβ^+^CD4^+^ donor cells). (**A**) Representative FACS plots of CD44 and CD62L expression on CD4+ T cells from chronic mice pre- and post-sorting. (**B and C**) Recipient mice were co-transferred with equivalent numbers (3×10^6^) of T_CM_ and CD4^+^CD44^+^CD62L^−^ T cells. Recipient mice were analyzed on day 3 post-challenge. *, p = 0.028. (**D–F**) Sorted cells were co-transferred at their physiological pre-transfer ratio (1–1.5×10^6^ T_CM_; 3–4×10^6^ CD4^+^CD44^+^CD62L^−^). At the indicated times post-infection cells from recipient mice were subjected to short-term (8 hours) Ag re-stimulation. (**D**) Representative dot-plots of IFN-γ production and dilution of VIOLET proliferation dye from a single-mouse. Numbers in dot plots represent frequencies. (**E**) Pooled analysis of the proliferation history of transferred, IFN-γ^+^, cells on day 5 p.i. Data is pooled from three independent experiments. ***, p = 0.005; **, p<0.008; *, p = 0.012. (**F**) Total number of transferred cells per ear over time. Data is pooled from three independent experiments. (**G**) Sorted cells were independently transferred into naïve recipient mice at their physiological ratio. Three weeks post challenge with 500 *L.m.* metacyclic promastigotes the ears and dLN were analyzed for parasite burden by limiting dilution analysis. *, p = 0.022 (ears) or p = 0.028 (dLN) versus the naïve group. Data is representative of two or more independent experiments.

Infection dependent polyclonal CD4^+^CD62L^−^ T cells from mice with a chronic primary infection have been shown to mediate protection following adoptive transfer and needle challenge [12]. We employed an adoptive transfer system that for the first time allowed tracking of CD4^+^CD44^+^CD62L^−^ cells to the dermal challenge site in recipient mice so that their recruitment, functionality, and lifespan could be assessed. Violet proliferation-dye labeled, CD4^+^CD44^+^CD62L^+^ T_CM_ or CD4^+^CD44^+^CD62L^−^ cells, which potentially contain both T_EM_ and T_EFF_ cells, were sorted from the spleens and dLNs of chronic mice and co-transferred into naïve recipients (Fig. 4A and Fig. S2). Prior to transfer both CD44^+^CD62L^+^ and CD44^+^CD62L^−^ Th cells were enriched for the Th1 markers T-bet and CXCR3, and the activation markers CD69, ICOS, and CD54 (ICAM-1) (Fig. S3A). Prior studies have shown IL-7R is rapidly re-expressed on effector CD4^+^ T cells after a period of non-expression during clonal expansion [17], [25], [26]. Therefore, IL-7R expression does not define a CD4^+^ T cell as a memory cell. Consistent with these observations we found both CD44^+^CD62L^+^ and CD44^+^CD62L^−^ Th cells were predominantly IL-7R high, but that the CD44^+^CD62L^−^ population contained a slightly higher frequency of IL-7R negative or low cells (Fig. S3B).

Direct comparison of the tissue homing potential of polyclonal CD44^+^CD62L^+^ and CD44^+^CD62L^−^ Th cells was analyzed following co-transfer of both populations at equivalent numbers and challenge with *L. major* the next day. On day 3 p.i., CD44^+^CD62L^−^ cells had homed preferentially to the ear without having undergone division while CD44^+^CD62L^+^ cells were found in the dLN but not the ear (Fig. 4B and 4C). While either population could be found in the spleen of naïve or challenged mice, *L.m.* inoculation was required to observe CD44^+^CD62L^−^ cells in the ear or the proliferation of CD44^+^CD62L^+^ cells in the dLN on day 4 p.i. (Fig. S4). Two-photon imaging of the ear dermis following challenge also revealed the presence of donor CD4^+^ T cells and the dynamic patrolling behavior of these cells was similar to what has been reported during the early primary response (Movie s1 and Movie s2) [27].

By day 5 p.i., antigen-specific IFN-γ^+^ T cells derived from either donor population could be found in the ear (Fig. 4D). However, the vast majority of IFN-γ^+^ cells from the CD44^+^CD62L^−^ population had not divided while the few IFN-γ^+^ cells derived from the CD44^+^CD62L^+^ T_CM_ population had virtually all divided (Fig. 4E). On day 12 p.i. the number of non-proliferated IFN-γ^+^CD44^+^CD62L^−^ donor cells was reduced to virtually zero, while a large and expanded population of proliferated IFN-γ^+^ CD44^+^CD62L^+^ T_CM_ derived cells was detected (T_CM_, D5 vs. D12 p = 0.028) (Fig. 4F). Similar to previous observations [12], [28], T_CM_ cells acquired effector function only after proliferating in the dLN (Figure S5). Despite the high proliferative capacity of T_CM_ cells and the resulting numeric advantage of T_CM_-derived cells in the ear at day 12 p.i. (CD44^+^CD62L^−^ vs. T_CM_, D12 p = 0.028), analysis of parasite loads in the ear and dLN of recipient animals receiving each population alone revealed that only CD44^+^CD62L^−^ cells conferred protection at 21 days p.i. (Fig. 4G). These observations directly correlate the early recruitment of IFN-γ-producing, non-dividing CD4^+^ T cells with protective immunity.

### Rapidly-recruited *L. major*-specific cells are T-bet^+^Ly6C^+^

**Figure 5:**
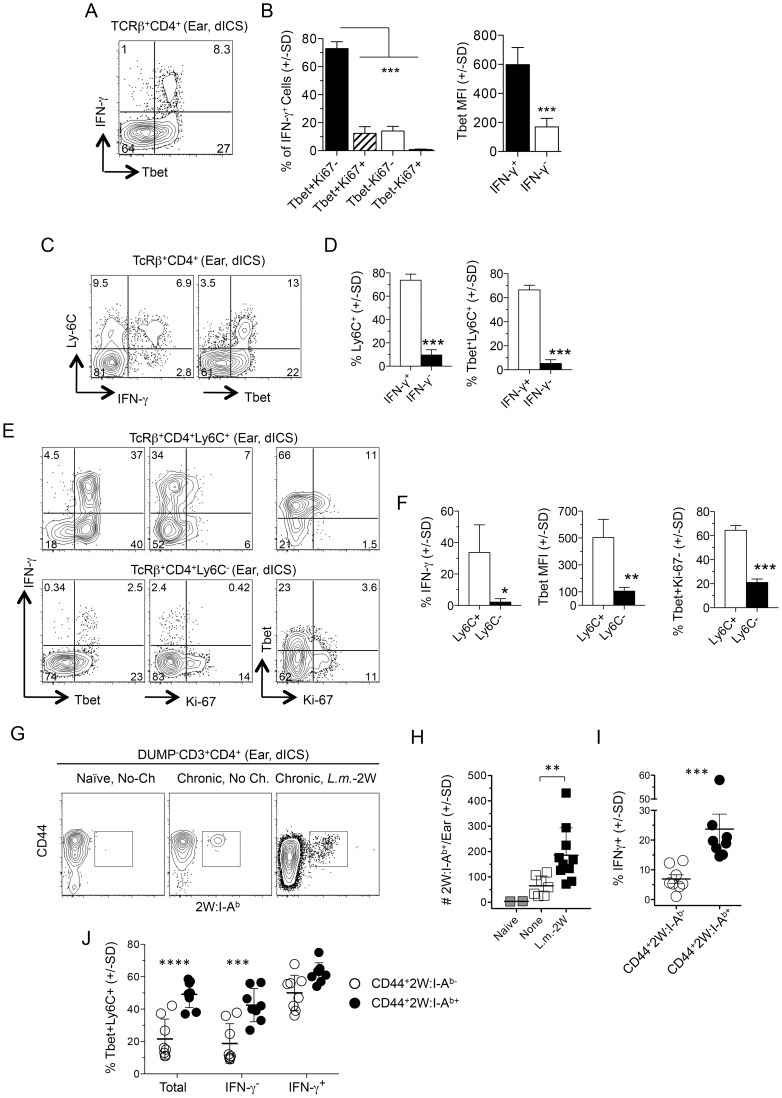
L.m.-specific cells express high levels of the differentiation markers T-bet and Ly6C at the site of challenge. (**A–F**) Uninfected ears of chronic mice were challenged by needle inoculation of 1×10^5^ *L.m.* 16–18 hours post-challenge TcRβ^+^CD4^+^ T cells from the ear were analyzed by flow-cytometry following dICS. (**A**) Representative dot plots of IFN-γ-production versus T-bet. (**B**) T-bet and Ki-67 expression by IFN-γ^+^ cells and average T-bet MFI by IFN-γ^+^ or IFN-γ^−^ cells from the same ear. ***, p≤0.0001, n = 4. (**C**) Representative dot-plot of Ly6C versus IFN-γ or T-bet expression. (**D**) The percentage of Ly6C^+^ cells and percentage of T-bet^+^Ly6C^+^ cells, within IFN-γ^+^ or IFN-γ^−^ cells from the same ear. ***, p<0.0001, n = 4. (**E**) Representative dot-plots of IFN-γ, T-bet, and Ki-67 expression by Ly6C^+^ or Ly6C^−^ cells. (**F**) The percentage of IFN-γ^+^ cells, average T-bet MFI, or the percentage of T-bet^+^Ki-67^−^ cells within Ly6C^+^ or Ly6C^−^ populations. *, p = 0.037; **, p = 0.010; ***, p<0.0001, n = 4. Data is representative of 4 independent experiments. (**G–J**) Analysis of 2W:I-A^b+^ parasite-specific cells in the ear. Naïve mice or mice with a chronic *L.m.*-2W infection in the contralateral ear were not challenged (No-Ch.) or challenged by needle inoculation of 1×10^5^ *L.m.*-2W (*L.m.*-2W). 3–4 days later CD4^+^ T cells from the ear were analyzed by flow-cytometry. Plots are gated on CD11b^−^CD11c^−^MHCII^−^F4/80^−^CD8^−^(DUMP^−^) CD3^+^CD4^+^ T cells. (**G**) Representative flow cytometry plot of CD44 versus 2W:I-A^b^ staining of cells from a single ear. (**H**) The number of 2W:I-A^b^-specific cells per ear. **, p = 0.003. (**I**) Percentage of IFN-γ^+^ χελλσ ωιτηιν 2W:I-A^b+^ or 2W:I-A^b−^ populations following dICS. ***, p = 0.0002. (**J**) The percentage of T-bet^+^Ly6C^+^ cells among total, IFN-γ^−^ or IFN-γ^+^ 2W:I-A^b^-specific or 2W:I-A^b^-non-specific cells in the ear following dICS. ****, p<0.0001; ***, p<0.001. Data is pooled from 3 (H) or 2 (I and J), independent experiments employing 0–1 (Naïve), 2–3 (None), or 2–4 (*L.m.*-2W) ears per experiment. Statistical comparisons are between 2W:I-A^b^-specific and 2W:I-A^b^-non-specific cells unless otherwise noted.

*Leishmania*-specific cells within the CD44^+^CD62L^−^ Th cell population are phenotypically heterogenous, and likely include both CD4^+^ T_EFF_ cells and T_EM_ cells. A high level of T-bet expression has been associated with a T_EFF_ phenotype [26]. On day 1 post-challenge of chronic mice dermal IFN-γ^+^ cells from the challenge site were highly enriched for T-bet^+^Ki-67^−^ cells (Fig. 5A and 5B) and were largely T-bet high versus IFN-γ^−^ T cells (Fig. 5B). Recently, LCMV-specific Ly-6C^+^ cells were identified as T-bet high Th1 T_EFF_ cells [26] that had a shorter lifespan than Ly6C^−^ cells, and expressed a gene expression profile associated with activation rather than memory. Early after challenge dermal-derived CD4^+^IFN-γ^+^ T cells were predominantly Ly6C^+^ and were highly enriched for T-bet and Ly6C co-expressing cells versus CD4^+^IFN-γ^−^ cells from the same tissue (Fig. 5C and 5D). Both IFN-γ^+^ and IFN-γ^−^ cells in the ear expressed low levels of CD27, a cell-surface molecule associated with memory cells [29], [30], and comparable levels of IL-7R (Figure S6). Regardless of IFN-γ-production, Ly6C was strongly co-expressed with T-bet (Fig. 5C) and Ly6C^+^ cells in the ear were highly enriched for IFN-γ^+^T-bet^+^Ki-67^−^ cells versus the Ly6C^−^ population (Fig. 5E and 5F).

We also employed a 2W peptide:MHC II tetramer that allows quantification of endogenous *L. major* specific cells following infection with transgenic *L. major*-2W [31] without relying on antigen driven, intracellular cytokine accumulation. In agreement with our dICS analysis of polyclonal antigen-specific IFN-γ^+^ cells, i.d. challenge of mice with a chronic *L. major*-2W infection resulted in a significant increase in 2W:I-A^b^ tetramer^+^ cells in the ear dermis by 3 days post-challenge (Fig. 5G and 5H). Note that a few of these CD4^+^ cells were found patrolling the skin even prior to challenge [22]. Direct ICS also revealed an enrichment of IFN-γ^+^ cells amongst the rapidly recruited 2W:I-A^b^ -specific population (Fig. 5I), of which the majority were Ly6C^+^T-bet^+^ cells (Fig. 5J). Ly6C^+^T-bet^+^ cells were also highly enriched in the total or IFN-γ^−^ 2W:I-A^b^-specific population versus the CD44^+^2W:I-A^b^ tetramer^−^ cells (Fig. 5J), demonstrating that even amongst non-IFN-γ-producing *L. major*-specific cells there is a significant enrichment of Ly6C^+^T-bet^+^ cells in the re-challenge site.

These data implicate the rapidly recruited *L. major*-specific cells defined in Figures 2–4 as Ly6C^+^T-bet^+^ T_EFF_ cells.

### Chronic *L. major* infection maintains high frequencies of antigen-specific Ly6C^+^ T_EFF_ cells

**Figure 6:**
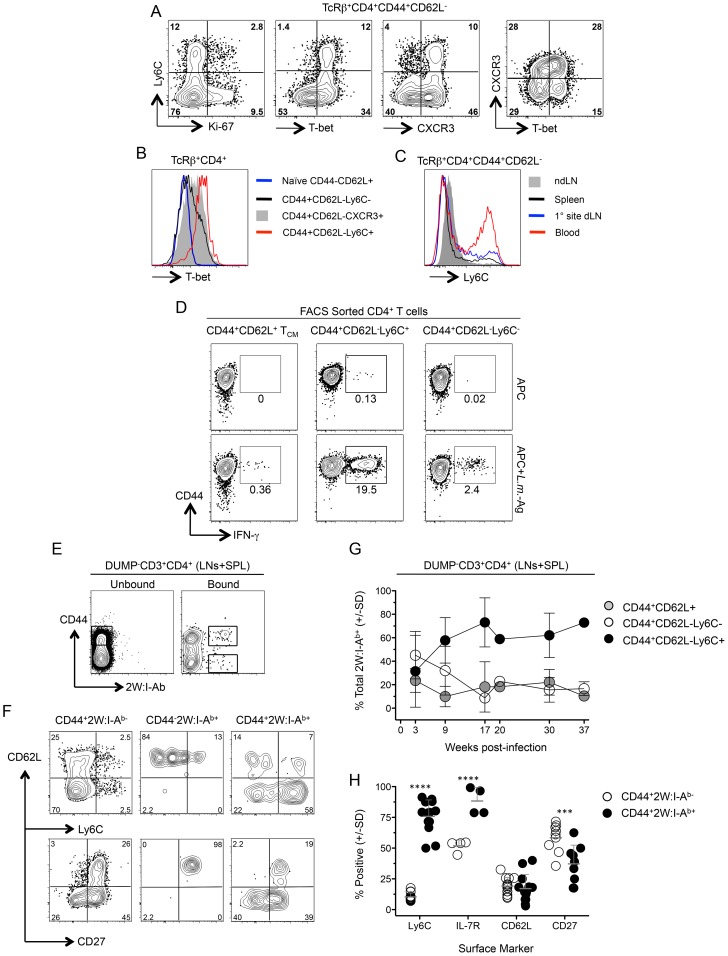
Ly6C^hi^T-bet^hi^ CD4^+^ T cells exist prior to challenge and are the predominant phenotype of antigen-specific cells during chronic infection. (**A**) Representative FACS plots of Ly-6C, T-bet, Ki-67 and CXCR3 expression by polyclonal TCRβ^+^CD4^+^CD44^+^CD62L^−^ cells from the spleens of chronic mice. (**B**) Analysis of T-bet expression by the indicated polyclonal T cell populations (**C**). Ly6C expression on polyclonal TCRβ^+^CD4^+^CD44^+^CD62L^−^ cells from the spleen, dLN, ndLN or blood of chronic mice. Data in (A–C) are representative of two independent experiments. (**D**) Representative flow cytometry plots of CD44 versus IFN-γ-staining of FACS sorted CD4^+^CD44^+^CD62L^−^Ly6C^+^, CD4^+^CD44^+^CD62L^−^Ly6C^−^ or CD4^+^CD44^+^CD62L^+^ T_CM_ CD3^+^CD4^+^ T cells from the spleen, dLN, ndLNs and blood of chronic mice following restimulation with APC alone or *L.m.*-Ag+APC. (**E–H**) 2W:I-A^b^-specific CD4^+^ T cells from the spleen, dLN and ndLNs of mice with a chronic primary *L.m.*-2W infection in the ear were magenetically enriched at the indicated time-points post-infection. Plots are gated on CD11b^−^CD11c^−^MHCII^−^F4/80^−^CD8^−^(DUMP^−^) CD3^+^CD4^+^ T cells. (**E**) Representative flow cytometry plot of CD44 versus 2W:I-A^b^ staining on cells in the bound (Bound) or nonbinding column flow through (Unbound) following magnetic enrichment of 2W:I-A^b^-specific cells. (**F**) Representative phenotypic analysis of the indicated populations at >9 weeks post-infection and gated as shown in (E). (**G**) Kinetic analysis of the phenotype of 2W:I-A^b^-specific cells at the indicated times post infection. n = 2–5 mice per time point pooled from 4 independent experiments. (**H**) Pooled phenotypic analysis of CD44^+^2W:I-A^b−^ or CD44^+^2W:I-A^b+^ CD4^+^ T cells from the data depicted in (G) at >9 weeks post-infection. ****, p<0.0001; ***, p<0.001.

Ly6C expression on Th cells at the site of *Leishmania* challenge could be the result of recruitment or antigen-exposure at the challenge site rather than a marker of the responding cells prior to challenge. Prior to challenge, the CD44^+^CD62L^−^Ly6C^+^ cells in the spleen of chronic mice had characteristics similar to those of dermal CD4^+^IFN-γ^+^Ly6C^+^ T cells following challenge, namely low Ki-67 expression and high expression of T-bet (Fig. 6A). Unlike CD44^+^CD62L^−^ cells positive for the canonical Th1 marker CXCR3, Ly6C^+^ cells were exclusively T-bet high (Fig. 6B), and were highly enriched in the circulation compared to the spleen, dLN, or ndLN (Fig. 6C). Prior to challenge, Ly6C^+^ and Ly6C^−^ polyclonal CD44^+^CD62L^−^ cells expressed low levels of CD103 and were predominantly IL-7R^+^, however, only the Ly6C^−^ population contained cells that also expressed CD27 (Fig. S7). In addition, a proportion of the Ly6C^+^ population in the dLN was proliferating or had recently proliferated based upon Ki-67 expression (Fig. S7B). Sorted CD44^+^CD62L^−^Ly6C^+^ T cells from chronic mice were also enriched for cells with the capacity to produce IFN-γ immediately upon antigen stimulation (Fig. 6D).

We recently employed the 2W peptide:MHC II tetramer to demonstrate that parasite-specific cells in the spleen and LNs expand during the first weeks of *L. major*-2W infection in the skin and then contract, maintaining constant numbers and function during the chronic stage of infection [31]. We extend these studies by showing 2W:I-A^b^ tetramer^+^ cells from the spleen and LNs were highly enriched for Ly6C^+^ cells versus the CD44^+^2W:I-A^b−^ population (Fig. 6, E–H). Analysis of the frequencies of the CD44^+^CD62L^−^Ly6C^+^, T_CM_ and CD44^+^CD62L^−^Ly6C^−^ subsets within the 2W:I-A^b^ tetramer+ cells over the course of infection revealed that CD44^+^CD62L^−^Ly6C^+^ T cells accounted for 60% of parasite-specific cells throughout the chronic stage of infection (9–37 weeks) (Fig. 6G). The 2W:I-A^b^ -specific CD4^+^ T cells were also enriched for IL-7R but not for CD62L, and decreased for expression of the memory marker CD27 during chronic infection (Fig. 6H). These data demonstrate that *L. major* infection preferentially maintains a high frequency of antigen specific CD44^+^CD62L^−^Ly6C^+^ cells during chronic infection and these cells have properties of circulating T_EFF_ cells.

### Ly6C^+^ T_EFF_ cells emulate the concomitant immune response

**Figure 7:**
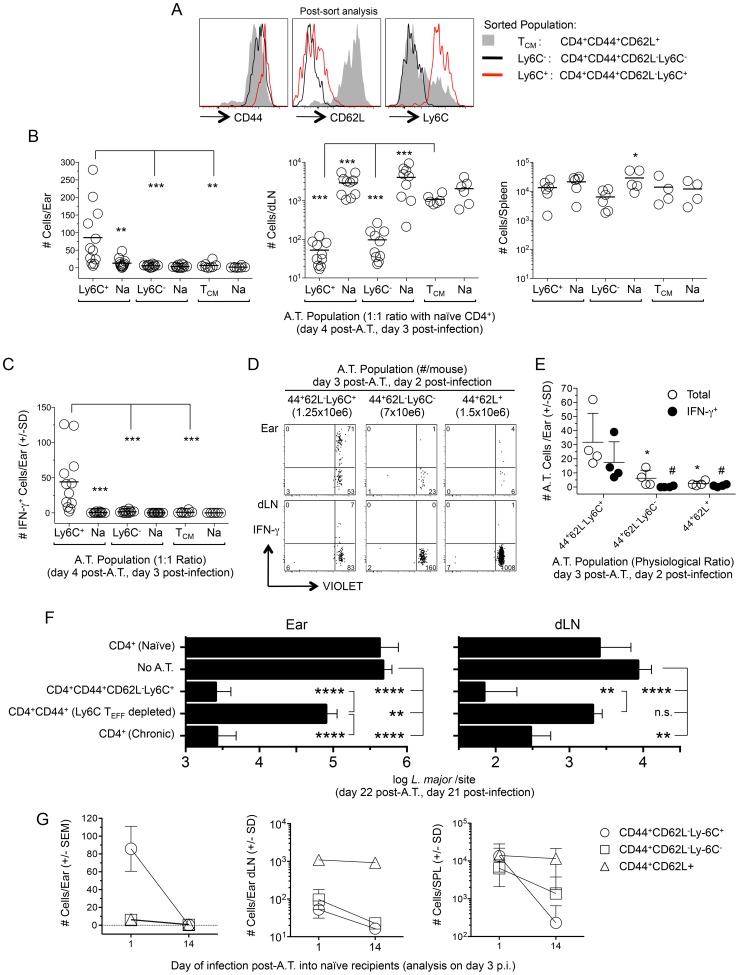
Preexisting Ly6C^+^ cells from chronic mice migrate to the site of challenge, produce cytokine and mediate protective immunity but are short-lived in the absence of infection. (**A–C**) Approximately equal numbers of FACS sorted CD44^+^CD62L^−^Ly6C^+^ (1.7–2.5×10^6^), CD44^+^CD62L^−^Ly6C^−^ (2–2.5×10^6^) or T_CM_ (1–2×10^6^) CD4^+^ T cells derived from the spleen, dLN, ndLNs and blood of chronic mice were labeled with a VIOLET proliferation dye and independently transferred with GFP^+^CD4^+^ T cells from naïve mice into naïve congenic recipients. Mice were challenged with 1×10^5^ *L.m.* on day 1 post-transfer and three days post-challenge ears or purified CD4+ T cells from the dLN or spleens of recipient mice were analyzed for the presence of transferred cells by flow cytometry following surface staining (dLN and spleen) or dICS (ears). (**A**) Representative post-sort analysis of the indicated FACS sorted populations. (**B**) Pooled analysis of the absolute number of cells from the indicated transferred population in recipient ears, dLN or spleen. In one experiment the dLNs from one mouse in each group was excluded due to a technical error. (**C**) The absolute number of IFN-γ^+^ cells per ear from the mice depicted in (B). (**D and E**) Sorted cells from chronic mice were adoptively transferred as in (B) while maintaining their pre-transfer physiological ratio (35 weeks p.i.) and transferred cells analyzed 2 days following challenge of congenic recipients employing dICS. (**D**) Representative concatenated dot plot of 2 ears or dLNs. Numbers in quadrants represent total number of cells. (**E**) Scatter plot of total cells and IFN-γ^+^ cells from individual ears of recipient animals following dICS. *(total) and ^#^(IFN-γ^+^), p<0.05, vs. CD44^+^CD62L^−^Ly6C^+^. (**F**) CD44^+^CD62L^−^Ly6C^+^ CD4^+^ T_EFF_ cells (3–3.4×10^6^) or CD44^+^CD4^+^ T cells depleted of the Ly6C^+^ T_EFF_ population (CD4^+^CD44^+^(Ly6C T_EFF_ depleted)) (5.7–5.8×10^6^) were FACS sorted from the spleen, dLN, ndLNs, and blood of chronic mice and independently transferred at their pre-transfer physiological ratios into naïve recipient mice. Separate groups of naïve recipient mice received purified CD4^+^ T cells from naïve (8.5–12×10^6^/mouse) or chronic (23–25×10^6^/mouse) mice. Ly6C^+^ T_EFF_ or CD4^+^CD44^+^(Ly6C T_EFF_ depleted) populations were co-transferred with purified naïve CD4^+^ cells to ensure the same approximate number of total cells was transferred to each mouse receiving chronic cells. On day 1 post-transfer recipient mice were challenged in both ears with 5×10^3^ *L.m.*-metacyclic promastigotes. On day 21 post-challenge the parasite load in the ear or dLN was determined by limiting dilution analysis. Data is pooled from two independent experiments with similar results totaling 10 (CD4^+^ Chronic), 10 (CD4^+^CD44^+^CD62L^−^Ly6C^+^), 14 (CD4^+^CD44^+^(Ly6C T_EFF_ depleted), or 14 (No A.T.) ears per group. Adoptive transfer of naïve cells was performed in 1 experiment (6 total ears) and did not significantly influence parasite load (p = 0.48). (**G**) Equivalent numbers of sorted cells were adoptively transferred into naïve recipients as described in (B). Half the mice from each recipient group were challenged with 1×10^5^ *L.m.* on day 1 post-transfer and the remaining half were challenged on day 14 post-transfer. Data is the absolute number of transferred cells recovered from the ear, dLN or spleen of recipient animals on day 3 post-challenge. Data in (B, C and G) is pooled from 4 independent experiments employing a total of 4 (T_CM_) or 6 (Ly6C− and Ly6C+ T_EFF_ populations) mice per time point. Ears, dLN and spleens were analyzed individually.

We next wished to formally demonstrate that pre-existing CD44^+^CD62L^−^Ly6C^+^ T_EFF_ cells are the source of rapidly recruited IFN-γ^+^ cells at the site of challenge. Approximately equal numbers of sorted CD44^+^CD62L^−^Ly6C^+^, CD44^+^CD62L^−^Ly6C^−^ or CD44^+^CD62L^+^ T_CM_ CD4^+^ T cells derived from chronic mice, see Fig. 7A, were independently transferred with GFP^+^CD4^+^ T cells from naïve mice to act as an internal control, into congenic recipients. Mice were challenged with *L. major* 1 day following transfer. Three days following challenge, ears, and purified CD4^+^ T cells from the spleen and ear dLNs of recipient mice were analyzed for the presence of adoptively transferred cells (Fig. 7B). CD44^+^CD62L^−^Ly6C^+^ cells preferentially homed to the ear following challenge as compared to both co-transferred CD4^+^ cells from naïve mice (p = 0.003) and CD44^+^CD62L^−^Ly6C^−^ (p = 0.002) or CD44^+^CD62L^+^ T_CM_ (p = 0.001) cells. In contrast, CD62L^+^ T_CM_ cells and naïve CD4^+^ T cells preferentially homed to the dLN as compared to either CD44^+^CD62L^−^Ly6C^+^ or CD44^+^CD62L^−^Ly6C^−^ cells. All populations were found in the spleen in similar numbers. Because the number of naïve GFP^+^ cells was the same in all recipients, we conclude that differences in the number and location of sorted chronic cells is due to functionality, and not mouse to mouse variation. Analysis of IFN-γ production in the ear employing dICS revealed an average of 44 adoptively transferred Ly6C^+^ cells were making IFN-γ *in-vivo* (Fig. 7C). Surprisingly, even when polyclonal CD44^+^CD4^+^ T cells from mice infected for 35 weeks were transferred at their pre-sort physiological ratios, see Figure 7D, CD44^+^CD62L^−^Ly6C^−^ cells were significantly under represented in the ear versus CD44^+^CD62L^−^Ly6C^+^ cells (p<0.05) and did not produce IFN-γ by dICS (Fig. 7, D and E). These data demonstrate that pre-existing Ly6C^+^ T_EFF_ cells within the CD44^+^CD62L^−^ population are the source of the *L. major*-specific CD4^+^ T cells that emulate the protective response against secondary challenge.

### Ly6C^+^ CD4^+^ T_EFF_ cells mediate strong protective immunity in the ear and ear dLN upon adoptive transfer

To determine if the Ly6C^+^ T_EFF_ cells are sufficient and necessary to confer optimal protection, we transferred polyclonal sort-purified CD4^+^CD44^+^CD62L^−^Ly6C^+^ T_EFF_ cells from chronic mice or the CD4^+^CD44^+^ T cells from chronic mice that were sort-depleted of the CD44^+^CD62L^−^Ly6C^+^ T_EFF_ population into naïve recipients at their pre-sort physiological ratios. Mice receiving purified CD4^+^ T cells from naive or chronic mice were employed as controls. Total CD4^+^ T cells from chronic mice were transferred in sufficient numbers so as to match the total number of CD44^+^ cells employed in the CD44^+^CD62L^−^Ly6C^+^ T_EFF_ and CD4^+^CD44^+^(Ly6C^+^ T_EFF_ depleted) adoptive transfers. One day following transfer, mice were challenged in the ear dermis with 5×10^3^ *L. major* parasites. Three weeks post-challenge, Ly6C^+^ T_EFF_ cells mediated a highly significant 204-fold reduction in parasite load in the ear (p<0.0001) and a 122-fold reduction in the dLN (p<0.0001) versus non-transferred control animals (Fig. 7F). The protection conferred by the Ly6C^+^ T_EFF_ population was indistinguishable from that conferred by the total CD4^+^ chronic population. By contrast, the CD44^+^CD4^+^ T cells depleted of the Ly6C^+^ T_EFF_ cells mediated only a 6.6-fold reduction in parasite load in the ear versus non-transferred controls (p = 0.009) that was 31-fold less than the protection conferred by the Ly6C^+^ T_EFF_ cells (p<0.0001), and no protection in the dLN. These data demonstrate that Ly6C^+^ T_EFF_ cells rapidly recruited to the site of *L. major* challenge mediate and are required for optimal protection.

### Ly6C^+^CD62L^−^CD44^+^CD4^+^ T cells from chronically infected mice are short lived in the absence of infection

Immunological memory, by definition, is mediated by a population of persistent memory cells that do not require the continued presence of the antigen that led to their generation [32]. Therefore, we wished to determine if CD4^+^CD44^+^CD62L^−^Ly6C^+^ T cells could be maintained in the absence of antigen. Sorted populations of CD44^+^CD62L^−^Ly6C^+^, CD44^+^CD62L^−^Ly6C^−^, or CD44^+^CD62L^+^ T_CM_ cells from chronic animals were labeled with a proliferation dye and transferred into naïve recipients at approximately equal numbers. Recipient mice were rested for either 1 or 14 days post-transfer and challenged with *L.major* in the ear dermis. The small number of cells that non-specifically proliferated during the 14-day rest period were excluded from analysis. On day 3 post-challenge, when antigen-induced proliferation is minimal (Figure 4B), ears, purified CD4^+^ T cells from the spleen and ear dLNs were analyzed for the presence of adoptively transferred cells (Figure 7G). As expected, recipient mice rested for 1 day contained CD44^+^CD62L^−^Ly6C^+^, but not CD44^+^CD62L^−^Ly6C^−^ or T_CM_ cells in the ear. In contrast, if challenge was delayed until 14 days post-transfer, no CD44^+^CD62L^−^Ly6C^+^ cells were detected in the ear (D1 vs. D14 p<0.0001), indicating these cells were no longer available for recruitment to the skin. Remarkably, CD44^+^CD62L^−^Ly6C^+^ cells, which were found in equivalent numbers with T_CM_ cells in the spleen when challenge occurred on day 1 post-transfer, were reduced 58-fold when challenge was delayed to day 14 (D1 vs. D14 p = 0.002), and were detected at significantly lower numbers than T_CM_ cells at this time (D14, T_CM_ vs. Ly6C^+^ p = 0.009). T_CM_ cells were detected in equal numbers in the dLN and spleen regardless of the time of challenge (dLN p = 0.54; SPL p = 1.0). Interestingly, CD44^+^CD62L^−^Ly6C^−^ cells displayed an intermediate, but significant (p = 0.02), five-fold drop in numbers in the spleen when mice were rested for 1 versus 14 days, suggesting this population may contain longer-lived T_EM_ cells, as previously shown [26]. Both Ly6C^+^ and Ly6C^−^ cells were found in smaller numbers in the dLN when challenge was delayed to day 14 post-transfer (dLN, D1 vs. D14, p≤0.004). Together, these observations confirm that CD44^+^CD62L^+^ T cells are true memory cells with the capacity to survive in the naïve recipients, whereas the CD44^+^CD62L^−^Ly6C^+^ cells, which are the cells required for protective immunity, are short-lived.

### The source of CD4^+^CD44^+^CD62L^−^Ly6C^+^Tbet^+^ T cells during chronic infection

**Figure 8:**
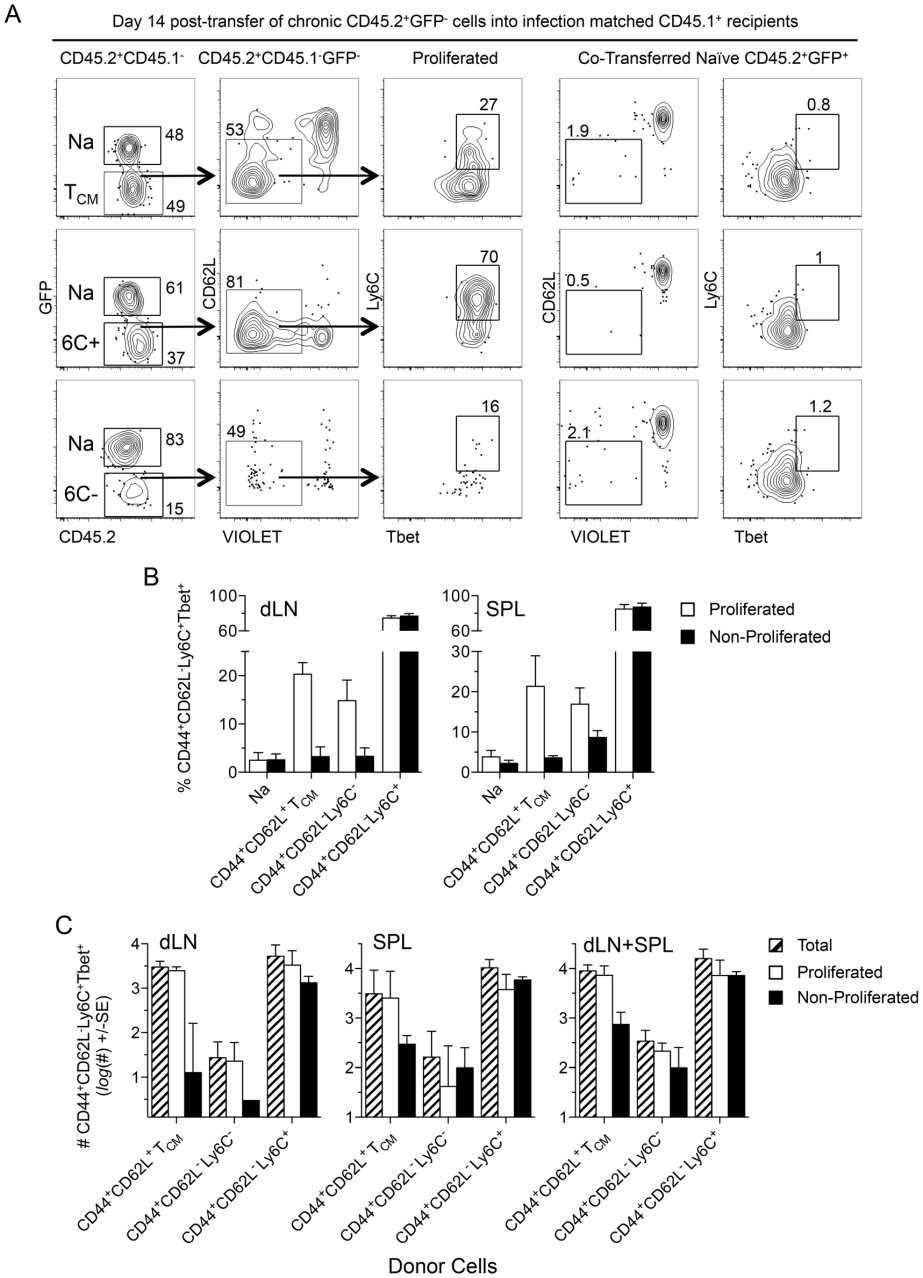
T_CM_ and CD44^+^CD62L^−^Ly6C^+^ cells generate CD44^+^CD62L^−^Ly6C^+^Tbet^+^ cells during chronic infection. (**A–D**) Equivalent numbers of FACS sorted cells as described in Figure 7 were adoptively transferred into infection matched congenic recipients. Fourteen days post-transfer purified CD4+ T cells from the dLN and spleens of recipient mice were analyzed for the presence of transferred cells by flow cytometry. (**A**) Representative concatenated dot plots of 2 dLNs gated on transferred cells. Numbers in quadrants represent frequency. (**B**) Analysis of the frequency of CD44^+^CD62L^−^Ly6C^+^Tbet^+^ cells within the proliferated or non-proliferated portion of the indicated transferred population. (**C**) Analysis of the absolute number of total, proliferated, or non-proliferated CD44^+^CD62L^−^Ly6C^+^Tbet^+^ cells per organ or per mouse (dLN+SPL) derived from the indicated transferred population. Data in (B) is pooled from 3 independent experiments employing a total of 4–6 mice per group. Data in (C) is pooled from 2 independent experiments in which counting beads were employed to determine the absolute number of cells employing a total of 3–4 mice per group.

In order to explain how such a high proportion of antigen experienced cells can be maintained as ‘short-lived’ T_EFF_ during chronic infection, we wished to investigate which T cell populations could give rise to these cells. Sorted populations of CD44^+^CD62L^−^Ly6C^+^, CD44^+^CD62L^−^Ly6C^−^, or CD44^+^CD62L^+^ T_CM_ T cells from chronic animals were labeled with a proliferation dye and co-transferred with dye-labeled naïve CD62L^+^GFP^+^CD4^+^ cells from naïve mice into infection-matched recipients. Fourteen days later transferred cells in the dLN and spleen were analyzed for Ly6C and Tbet expression (Figure 8). T_CM_ cells underwent robust proliferation whereas co-transferred CD62L^+^ naïve GFP^+^CD4^+^ T cells did not (Figure 8A, top panels). Analysis of proliferated T_CM_ derived cells revealed a significant increase in the frequency of CD44^+^CD62L^−^Ly6C^+^Tbet^+^ T_EFF_ cells versus non-proliferated T_CM_ cells in both the dLN (p = 0.005) and spleen (p = 0.036) and the same was true for CD44^+^CD62L^−^Ly6C^−^ cells in the dLN (p = 0.004) (Figure 8B). Remarkably, a proportion of transferred CD44^+^CD62L^−^Ly6C^+^ cells, which do not need to undergo proliferation to mediate effector function, also underwent proliferation in infection-matched recipients and maintained Ly6C and Tbet co-expression in both the dLN and spleen (Fig. 8A, middle panels, and Fig. 8B). Although the frequency of Ly6C^+^Tbet^+^ cells was considerably lower among proliferated T_CM_ versus CD44^+^CD62L^−^Ly6C^+^ derived cells in both the spleen and dLN (p<0.0001), the numerical superiority of the total T_CM_ population (Figure S8) resulted in a non-significant difference in the number of proliferated or total CD44^+^CD62L^−^Ly6C^+^Tbet^+^ cells derived from either population at 14 days post-transfer in the dLN, spleen, or dLN+spleen (Fig. 8C). In contrast to the low number of CD44^+^CD62L^−^Ly6C^+^ cells found in naïve mice on day 14 post-transfer (Figure 7G), substantial numbers of non-proliferated CD44^+^CD62L^−^Ly6C^+^ cells expressing Tbet were found 14 days following transfer into infection-matched recipients. While CD44^+^CD62L^−^Ly6C^+^Tbet^+^ cells derived from the CD44^+^CD62L^−^Ly6C^−^ population were also found, the total number of these cells was significantly less than that observed in mice receiving T_CM_ (p = 0.003) or CD44^+^CD62L^−^Ly6C^+^ cells (Fig. 8C, right panel, p = 0.001). These observations demonstrate that re-exposure of *Leishmania*-specific cells to antigen during chronic infection can lead to the generation of CD44^+^CD62L^−^Ly6C^+^Tbet^+^ cells, thereby contributing to the high frequency of this population prior to challenge.

## Discussion

The protective immunity induced by a healed but persistent primary infection remains the ‘gold standard’ of acquired resistance against *Leishmania major* infection in both mice and humans, and emulating this response remains a key objective of vaccines against all forms of Leishmaniasis. The current studies provide the most detailed analysis to date of the cells mediating this protective response. We found that rapidly recruited (within hours), pre-existing Th1 Ly6C^+^ T_EFF_ cells mediate immunity at the site of secondary *L. major* challenge in mice with a chronic primary infection. These findings, along with our previous observation that rapid immunity is a hallmark of protection against infected sand fly challenge in mice [11], [18], strongly suggest that natural immunity in individuals with a chronic primary infection against subsequent infection is dependent upon the presence of pre-existing CD4^+^ T_EFF_ rather than memory cells.

We employed an intra-dermal ear challenge model that allowed tracking of T_EFF_ cell subsets to the peripheral challenge site. So far as we are aware, this has not been achieved for *Leishmania* or any other pathogen. We also employed Ly6C [26], [33], a T-bet regulated GPI-anchored surface glycoprotein to further define CD44^+^CD62L^−^CD4^+^ T cells. A recent study also used the differential expression of Ly6C to distinguish between effector and memory Th1 CD4^+^ T cells responding to acute infection with LCMV, with Ly6C^lo^T-bet^int^ and Ly6C^hi^T-bet^hi^ cells defining longer-lived memory and shorter-lived effector populations, respectively [26]. The enrichment of pre-existing Ly6C^+^ T_EFF_ cells in peripheral blood, their rapid and non-specific homing to sites of tissue damage, their behavior as cells that rapidly secrete cytokine upon antigen encounter in the periphery without undergoing division following challenge, and particularly their short life span are traits that embody differentiated T_EFF_ rather than memory cells. In addition, Ly6C^+^ T_EFF_ cells were also CD27^−^, a phenotype associated with short-lived tissue homing cells [29], [30]. Despite their relatively short life span following transfer into naïve recipients, Ly6C^+^ T_EFF_ cells were the predominant antigen-specific CD4^+^ T cell population during chronic infection. Transfer of either T_CM_ or Ly6C^+^ T_EFF_ into infection-matched recipients led to the generation of Ly6C^+^Tbet^+^ T_EFF_ cells in the spleen and dLN, suggesting that each of these populations can contribute to the maintenance of the high frequency of Ly6C^+^ T_EFF_ cells during chronic infection. The continuous renewal of Ly6C^+^ T_EFF_ cells from thymic precursors [34] or from the activation of cells in a self-renewing pool as we observed here and has been reported by others [9], [35] may explain how the Ly6C^+^ T_EFF_ population escapes exhaustion that is typically associated at the single-cell level with chronic antigen stimulation [36].

The proliferative capacity of the Ly6C^+^ T_EFF_ cells in the infection-matched recipients was surprising since they disappeared during the same two weeks time frame in naïve recipients. The findings suggest that while Ly6C expression is indicative of an antigen-dependent effector cell, it does not imply that a cell is terminally differentiated. Rather, Ly6C^+^ cells may be recently activated effector cells that still retain proliferative capacity and can expand the pool of Ly6C^+^ T_EFF_ cells when re-exposed to antigen in a chronic infection setting. We also found significant numbers of non-proliferated Ly6C^+^ T_EFF_ cells in the dLN and spleen of infection-matched recipients, suggesting that their lifespan can be prolonged without undergoing division in the presence of infection. The proliferative capacity and antigen-dependency of the Ly6C^+^ population suggests these cells are recently activated effector cells, not infection-independent T_EM_ cells.

In mice infected with the *dhfr-ts^−/−^ L. major* mutant parasite, which is unable to establish chronic infection, only CD62L^+^CD4^+^ T_CM_ cells were maintained after parasite clearance and these cells mediated a compromised and delayed immune response upon needle challenge [12]. Akin to the failure of non-living vaccines in human field trials [20], the live mutant vaccine and multiple formulations of non-living vaccines have consistently underperformed the immunity against needle challenge established in chronic mice [11], [12], [18]. Antigen vaccines are especially ineffective against infected sand fly challenge due, we believe, to their inability to maintain cells that can be rapidly recruited and thereby counteract the early immunosuppressive conditions at the site of parasite delivery by sand fly bite [11], [18], [37]–[39]. Indeed, the marginal level of immunity transferred by the CD44^+^ population depleted of CD44^+^CD62L^−^Ly6C^+^ T_EFF_ cells is reminiscent of the compromised immunity conferred by non-persisting antigen vaccines following needle challenge and may in fact be due to cells that are already transitioning to Ly6C^+^ T_EFF_ cells just prior to transfer (Figure 8). Thus, while T_CM_ cells can eventually give rise to T_EFF_ cells, the late arrival of these cells to the inoculation site abrogates their efficacy.

The key contribution of pre-existent, short-lived Ly6C^+^ T_EFF_ cells to the protective response is the likely reason for the critical failure of non-living vaccines against cutaneous leishmaniasis and possibly other chronic infectious diseases for which immunological memory may be an insufficient condition of concomitant immunity [3]–[8], [40]. The primary role of Th1 T_CM_ cells may not be the generation of effector cells *following* secondary challenge, but rather, the generation of effector cells *prior* to secondary challenge via re-exposure to antigen provided by the chronic infection. If vaccination is able to generate a stable CD4^+^ memory population it may be possible to employ boosting or long-term antigen depots to generate the pre-existing T_EFF_ population necessary for protective immunity.

## Materials and Methods

### Mice

Female C57BL/6, B6.SJL-*Ptprc^a^ Pepc^b^*/BoyJ and C57BL/6-Tg(UBC-GFP)30Scha/J mice were obtained from Jackson Laboratories. Female C57BL/6 and B6.SJL-Cd45a(Ly5a)/Nai were obtained from Taconic Farms. All mice were maintained in the National Institute of Allergy and Infectious Diseases animal care facility under specific pathogen-free conditions (Animal Study Protocol LPD-68E).

### Leishmania parasites and infection of mice

*Leishmania major* Friedlin (FV1) and *L. major* RYN were obtained and grown as described previously [41]. *L. major* Friedlin-RFP [38] and *L.m.* Friedlin expressing the 2W peptide [31] were generated as described previously. Infective-stage metacyclic promastigotes were isolated from stationary cultures (4–6 day-old) by negative selection of non-infective forms using peanut agglutinin (PNA, Vector Laboratories Inc) [42].

Mice with chronic primary infections (Chronic mice) were generated by infection with 1×10^4^ *L. major* metacyclic promastigotes subcutaneously (s.c.) in the footpad, and used 10–20 weeks later, unless otherwise indicated, when footpad lesions had completely resolved. *L.m.*-2W chronic infections were established following inoculation with 1×10^5^ metacyclic promastigotes in the contralateral ear using a 27.5 gauge needle in a volume of 5–10 µl. For secondary needle challenge, mice were inoculated with the indicated number and strain of *L. major* metacyclic promastigotes in the ear in a volume of 5–10 µl. Transmission of *L. major*-RYN by exposure to the bites of infected female *Phlebotomus duboscqi* sand flies was carried out as described previously [41]. Mouse ears were exposed to the bites of 4–8 infected flies.

Parasite load per ear or dLN was determined by limiting dilution analysis as described previously [11].

### Sample preparation

Ear, dLN and spleen tissue was prepared as previously described [11]. Briefly for ears, the ventral and dorsal sheets were separated, deposited in 1 ml DMEM containing 160 µg/ml of Liberase TL purified enzyme blend (Roche Diagnostic Corp.), and incubated for 1.5–2 hours at 37°C and 5% CO_2_. In experiments employing dICS, 20 µg/ml of Brefeldin A was added and media pre-warmed to 37°C. Digested ear sheets were homogenized using the Medicon/Medimachine tissue homogenizer system (Beckton Dickinson). The ear tissue homogenate was then flushed from the medicon with 10 ml RPMI media containing 0.05% DNAse and filtered using a 50 um-pore-size cell strainer and placed on ice. In experiments employing dICS, 2 µg/ml of Brefeldin A was added to DNase media pre-warmed to 37°C and the ear homogenate returned to 37°C and 5% CO_2_ for an additional 1.5–2 hours. In some experiments, red blood cells were removed from spleen preparations using ACK lysing buffer (Lonza). Blood was obtained by intra-cardiac bleed and lymphocytes were purified using Isopaque-1077 (Sigma).

### Purification of CD4^+^ cells and pMHCII tetramer staining and magnetic enrichment

Purified CD4+ T cells were obtained from spleen or dLN using magnetic bead separation (Miltenyi Biotec). 2W:I-Ab tetramer staining and magnetic enrichment were performed as previously described [31]. Briefly, a single cell suspension of the spleen, dLN, and ndLNs from a single mouse were stained with 10 nM allophycocyanin- or phycoerythrin-labeled 2W:I-Ab-streptavidin tetramers for 1 hour at room temperature. Samples were then chilled to 4°C and incubated with magnetic anti-fluorochrome beads and run through a magnetized LS column (Miltenyi Biotec) in a 4°C cold room. To determine the absolute number of cells, a portion of each sample was removed for counting with AccuCheck Counting Beads (Invitrogen) as described previously [31].

### Re-stimulation of T cells

Single-cell suspensions were re-stimulated at 37°C in 5% CO_2_ for 8 (indicated transfer experiments) or 14 (overnight) total hours in flat-bottom 48-well plates with 0.5–1×10^6^ T cell-depleted (Miltenyi Biotech), irradiated, naïve spleen cells (APCs), with or without 50 µg/ml freeze-thaw *Leishmania* antigen (*L.m.*-Ag). During the last 4 hours of culture, 1 µg/ml of Brefeldin A (Golgiplug; BD Biosciences) was added. APC genotype was matched to recipient animals in adoptive transfer experiments. Following culture, washed cells were labeled with VIOLET or AQUA fixable Live/Dead dye (Invitrogen) to exclude dead cells.

### Analysis of cells by flow cytometry

All antibodies were from eBioscience or BD-Biosciences unless otherwise noted. Cells were stained with anti-Fc-γ III/II (CD16/32) receptor Ab (2.4G2, BD Biosciences) with or without VIOLET or AQUA fixable LIVE/DEAD dye (Invitrogen) in PBS containing 0.5–1.0% FCS for 10 minutes followed by incubation for 25 minutes with a combination of the following conjugated antibodies: Pacific Blue-, eFluor 450-, or allophycocyanin-eFluor-780-anti-B220 (RA3-6B2), anti-CD11b (MI-70), anti-CD11c (N418), anti-F4/80 (BM8), and/or anti-CD8α (53-6.7); FITC- or PerCP-Cy5.5-anti-Ly6C (HK1.4); V500- or V450-anti-CD3ε (145-2C11); PerCP-Cy5.5-, AlexaFluor 750-, V450-, or PE-Cy7-anti-CD4 (RM4-5); V500-, FITC-, or allophycocyanin-eFluor-780-anti-CD44 (IM7); allophycocyanin- or PE-Cy7-anti-CD127 (A7R34) or anti-CD27 (LG.7F9); allophycocyanin-, PE-Cy7-, or PE-anti-CD62L (MEL-14); Brilliant violet 421-anti-TCRβ (H57-597, Biolegend); PE-anti-CXCR3 (CXCR3-173), PE-anti-CD54(ICAM-1) (3E2), Per-CP Cy5.5-anti-CD69 (H1.2F3); FITC-anti-ICOS (C398.4A). In some experiments, samples were treated with the Foxp3 Fixation/Permeabilization Buffer (eBioscience) per manufacturer's instructions, and then stained with FITC-, or PE-anti-Ki-67 (B56); PE- or eFluor 660-anti-T-bet (eBio4B10); FITC-, PE-, eFluor450-, or allophycocyanin-anti-IFN-γ (XMG1.2), FITC-, PE- or Alexafluor-700 anti-TNF-α (MP6-XT22 or TN3-19); APC-, or Pacific Blue-anti-IL-2 (JES6-5H4). In some experiments, samples were fixed and permeabilized with BD Cytofix/Cytoperm (Becton-Dickinson) according to the manufacturer's instructions, and subsequently stained for 45″-1 hour at 4°C with a combination of the above mentioned anti-cytokine antibodies. Samples were run on LSRII or FACS Canto II flow cytometers (Becton-Dickinson) using FACS DIVA software. Forward-scatter and side-scatter width was employed to exclude cell doublets from analysis. Data was analyzed with FlowJo (Tree Star). Gating was determined by employing isotype controls and/or comparison with the relevant control population.

### Cell sorting and adoptive transfer

Pooled single cell suspensions from the spleen, dLN, ndLNs, and in some experiments blood, of chronic animals were stained with a combination of CD44, CD62L, CD4, TCRβ, CD11b, MHC II, NK1.1 and/or Ly6C, as described in the text and purified using FACsAriaIIu (BD Biosciences) cell sorters at the Flow Cytometry Section of the Research Technologies Branch of the NIAID. In some experiments, sorted populations were re-suspended at 1×10^6^ cells/ml in PBS+0.1% FBS at 37°C and labeled with 1 µM VIOLET proliferation dye (Invitrogen) for 14″ at RT. The reaction was stopped with neat FBS, and the cells were washed twice with cold PBS. Sorted populations were transferred independently by intravenous injection into congenic recipients, or, in some experiments, different sorted populations from C57BL/6 or B6.SJL mice were co-transferred into C57BL/6-Tg(UBC-GFP) recipient mice or a derivation thereof. The number of transferred cells is indicated in the text. Recipient mice were challenged one or 14 days following transfer. Congenic mice were source matched.

### Statistics

Comparison of cell numbers was done using the Mann-Whitney test. Comparisons of frequency were performed using the unpaired students t-test for comparisons between two groups or a one-way analysis of variance (ANOVA) with Bonferroni's post-test for comparisons between multiple groups. Comparison of cell numbers or parasite loads under conditions of proliferation was performed on *log*-transformed data using ANOVA. All p-values are two-sided. Statistical calculations were done in Graphpad PRISM 5.0c (www.graphpad.com). Level of significance is reported in the text.

### Ethics statement

All animal experiments were performed under the LPD-68E Animal Study Protocol approved by the NIAID Animal Care and Use Committee using guidelines established by the Animal Welfare Act and the PHS Policy on Humane Care and Use of Laboratory Animals.

## Supporting Information

Figure S1 **Analysis of cytokine production by direct intracellular staining (dICS) following needle challenge.** Ears of chronic (A and B) or naïve (B) mice were not challenged, needle inoculated with PBS or needle inoculated with 1×10^5^ *L.m.* metacyclic promastigotes (*L.m.*). 17–20 hours post-challenge, CD3^+^CD4^+^ T cells from the ear were analyzed by flow-cytometry following dICS, which stains for intracellular cytokines directly ex-vivo without the use of antigen or pharmacological stimulation. Two independent experiments are shown.
(PDF)

Figure S2 **Representative gating strategy for detection of co-transferred, sorted cells from chronic congenic mice in naïve UB-gfp recipients.** Age-matched female CD45.1 and CD45.2 mice were infected in the LHFP with *L. major*. 16–20 weeks later mice were sacrificed and cells sorted from the spleens, dLN, and ndLNs. Different sorted populations were labeled with a proliferation dye and co-transferred into naïve CD45.2 UB-gfp recipients. One day later mice were infected in the ear dermis with *L. major*. Following infection cells were analyzed by flow cytometry according to the depicted gating strategy. Inter- and intra-ear comparisons of different sorted populations generated similar results.
(PDF)

Figure S3 **Phenotypic analysis of polyclonal CD3^+^CD4^+^ T cells from mice chronically with** ***L. major*** **.** Naïve (CD44^−^CD62L^+^, black line), T_CM_ (CD44^−^CD62L^+^, red line), or T_EFF_/T_EM_ CD44^+^CD62L^−^ CD4^+^ T cells (blue line) from chronic mice were analyzed for expression of the indicated markers. (**B**) Analysis of the frequency of IL-7R negative/low cells or IL-7R MFI within the indicated populations. p<0.0001, n = 5.
(PDF)

Figure S4 **Homing of CD44^+^CD62L^−^ T_EFF_/T_EM_ cells to the ear and proliferation of CD44^−^CD62L^+^ T_CM_ cells in the lymph nodes does not occur in naïve recipient mice.** CD4^+^CD44^+^CD62L^+^ (T_CM_) and CD4^+^CD44^+^CD62L^−^ T cells were FACS sorted from chronic congenic mice, labeled with VIOLET proliferation dye and co-transferred into naïve UB-gfp mice (see Fig. S2). Recipient mice were not challenged (Naïve) or challenged with *L. major* one day post-adoptive transfer (*L.m.* day 1 post-A.T.). Adoptively transferred CD4^+^ T cells from the ear, dLN and spleen (SPL) of recipient mice were analyzed by flow cytometry on day 4 post-infection (day 4 p.i.). Representative dot-plot gated on GFP^−^TCRβ^+^CD4^+^ donor cells.
(PDF)

Figure S5 **Central memory CD4^+^ T cells acquire the capacity to produce IFN-γ only after expansion in the dLN.** (**A**) CD4^+^CD44^+^CD62L^+^ (T_CM_) and CD4^+^CD44^+^CD62L^−^ T cells were FACS sorted from chronic congenic mice, labeled with VIOLET proliferation dye and co-transferred into naïve UB-gfp mice (see Fig. S2). One day post-transfer recipient mice were challenged with *L. major*. Adoptively transferred CD4^+^ T cells from the dLN of recipient mice were analyzed at the indicated time-points post-challenge by flow cytometry. Representative dot-plot gated on GFP^−^TCRβ^+^CD4^+^ donor cells. (**B**) T_CM_ cells from chronic mice were transferred into naïve congenic recipient mice and analyzed for CD62L expression on day 12 post-infection, day 13 post-adoptive transfer.
(PDF)

Figure S6 **Phenotypic analysis of IFN-γ^+^ versus IFN-γ^−^ polyclonal CD3^+^CD4^+^ T cells in the ear of chronic mice following challenge.** (**A and B**) Uninfected ears of mice with a chronic infection in the footpad were challenged by needle inoculation with 2×10^5^ *L. major*. 20 hours post-challenge CD3^+^CD4^+^ T cells from the ear were analyzed by flow-cytometry employing dICS. (**A**) Representative histograms of IL-7R, CD27, and Ly6C expression on IFN-γ^+^ (blue line) versus IFN-γ^−^ (red line) cells from the dermal site of challenge, gated as shown in Figure S1. (**B**) Analysis of the median fluorescent intensity of the indicated surface markers on IFN-γ^+^ versus IFN-γ^−^ CD3^+^CD4^+^ T cells. ***, p<0.001, n = 4 ears.
(PDF)

Figure S7 **Phenotypic analysis of polyclonal CD3^+^CD4^+^ T cells from chronic mice.** Naïve CD44^−^CD62L^+^ (black line), CD44^+^CD62L^+^Ly6C^+^ (red line), or CD44^+^CD62L^−^Ly6C^−^ (blue line) T cells from chronic mice were analyzed for expression of the indicated markers. (**B**) Representative dot plot of Ki-67 expression versus IL-7R expression on the indicated populations in the dLN of chronically infected mice.
(PDF)

Figure S8 **Total cell recovery of T_CM_, CD44^+^CD62L^−^Ly6C^+^, or CD44^+^CD62L^−^Ly6C^−^ derived cells 14 days following transfer into infection matched congenic recipients.** Equivalent numbers of FACS sorted cells were adoptively transferred into infection matched congenic recipients as described in Figure 8. Fourteen days post-transfer purified CD4+ T cells from the dLN and spleens of recipient mice were analyzed for the presence of transferred cells by flow cytometry. Analysis of the absolute number of total, proliferated, or non-proliferated cells per organ or per mouse (dLN+SPL) derived from the indicated transferred population.
(PDF)

Movie S1 **Dynamic imaging of tissue resident cells derived from adoptively transferred CD4^+^CD44^+^CD62L^+^GFP^+^ T_CM_ cells following challenge with** ***L. major*** **-RFP.** GFP-expressing cells are shown in green, the 2-photon second harmonic signal is shown in blue, and *L. major*-RFP is shown in red. Playback speed is 600×. Scale bar, 50 µm. Two photon intravital imaging and image analysis was performed as described previously [11], [38]. Raw imaging data were processed with Imaris (Biplane) using a Gaussian filter for noise reduction. All images are displayed as 2D maximum intensity projections. Movie files of 3-dimentional images were generated using Imaris.
(MOV)

Movie S2 **Dynamic imaging of tissue resident CD4^+^CD44^+^CD62L^−^GFP^+^ T cells following adoptive transfer and challenge with** ***L. major*** **-RFP.** GFP-expressing cells are shown in green, the 2-photon second harmonic signal is shown in blue, and *L. major*-RFP is shown in red. Playback speed is 600×. Scale bar, 50 µm. Two photon intravital imaging and image analysis was performed as described previously (Peters et al., 2008; Peters et al., 2009). Raw imaging data were processed with Imaris (Biplane) using a Gaussian filter for noise reduction. All images are displayed as 2D maximum intensity projections. Movie files of 3-dimentional images were generated using Imaris.
(MOV)
