# Supplementary material for: Chronic Parasitic Infection Maintains High Frequencies of Short-Lived Ly6C+CD4+ Effector T Cells That Are Required for Protection against Re-infection
Source: PLoS Pathog. 2014 Dec 4;10(12):e1004538. doi: 10.1371/journal.ppat.1004538 (PMC4256462; doi:10.1371/journal.ppat.1004538)
Supplement: Figure S1 — Analysis of cytokine production by direct intracellular staining (dICS) following needle challenge. Ears of chronic (A and B) or naïve (B) mice were not challenged, needle inoculated with PBS or needle inoculated with 1×10^5^ L.m. metacyclic promastigotes (L.m.). 17–20 hours post-challenge, CD3+CD4+ T cells from the ear were analyzed by flow-cytometry following dICS, which stains for intracellular cytokines directly ex-vivo without the use of antigen or pharmacological stimulation. Two independent experiments are shown. (PDF) [file ppat.1004538.s001.pdf]

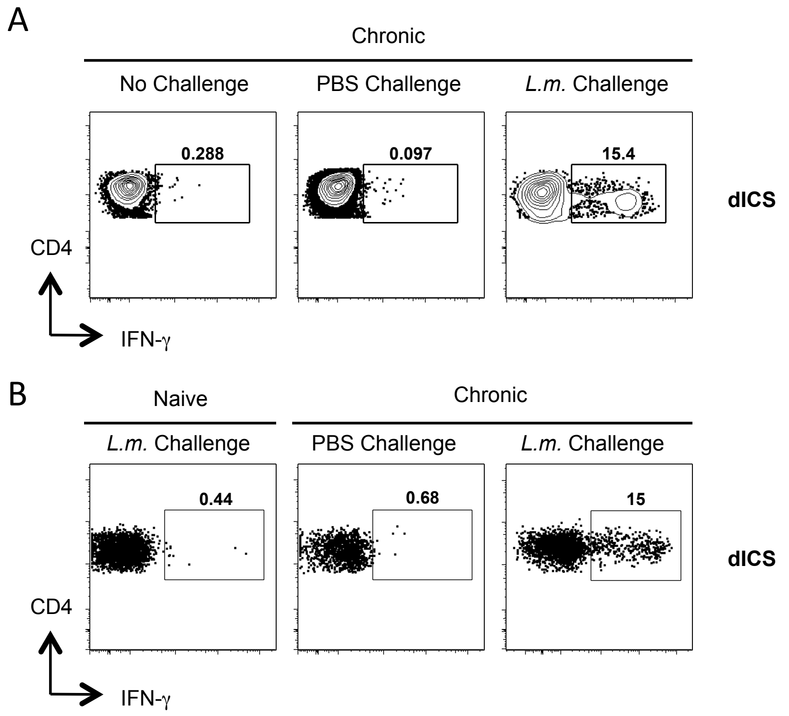

Figure S1. Analysis of cytokine production by direct intracellular staining (dICS) following needle challenge.

Ears of chronic (A and B) or naïve (B) mice were not challenged, needle inoculated with PBS or needle inoculated with $1x10^5$ *L.m.* metacyclic promastigotes (*L.m.*). 17-20 hours post-challenge, $CD3^+CD4^+$ T cells from the ear were analyzed by flow-cytometry following dICS, which stains for intracellular cytokines directly ex-vivo without the use of antigen or pharmacological stimulation. Two independent experiments are shown.
