# Supplementary material for: Chronic Parasitic Infection Maintains High Frequencies of Short-Lived Ly6C+CD4+ Effector T Cells That Are Required for Protection against Re-infection
Source: PLoS Pathog. 2014 Dec 4;10(12):e1004538. doi: 10.1371/journal.ppat.1004538 (PMC4256462; doi:10.1371/journal.ppat.1004538)
Supplement: Figure S2 — Representative gating strategy for detection of co-transferred, sorted cells from chronic congenic mice in naïve UB-gfp recipients. Age-matched female CD45.1 and CD45.2 mice were infected in the LHFP with L. major. 16–20 weeks later mice were sacrificed and cells sorted from the spleens, dLN, and ndLNs. Different sorted populations were labeled with a proliferation dye and co-transferred into naïve CD45.2 UB-gfp recipients. One day later mice were infected in the ear dermis with L. major. Following infection cells were analyzed by flow cytometry according to the depicted gating strategy. Inter- and intra-ear comparisons of different sorted populations generated similar results. (PDF) [file ppat.1004538.s002.pdf]

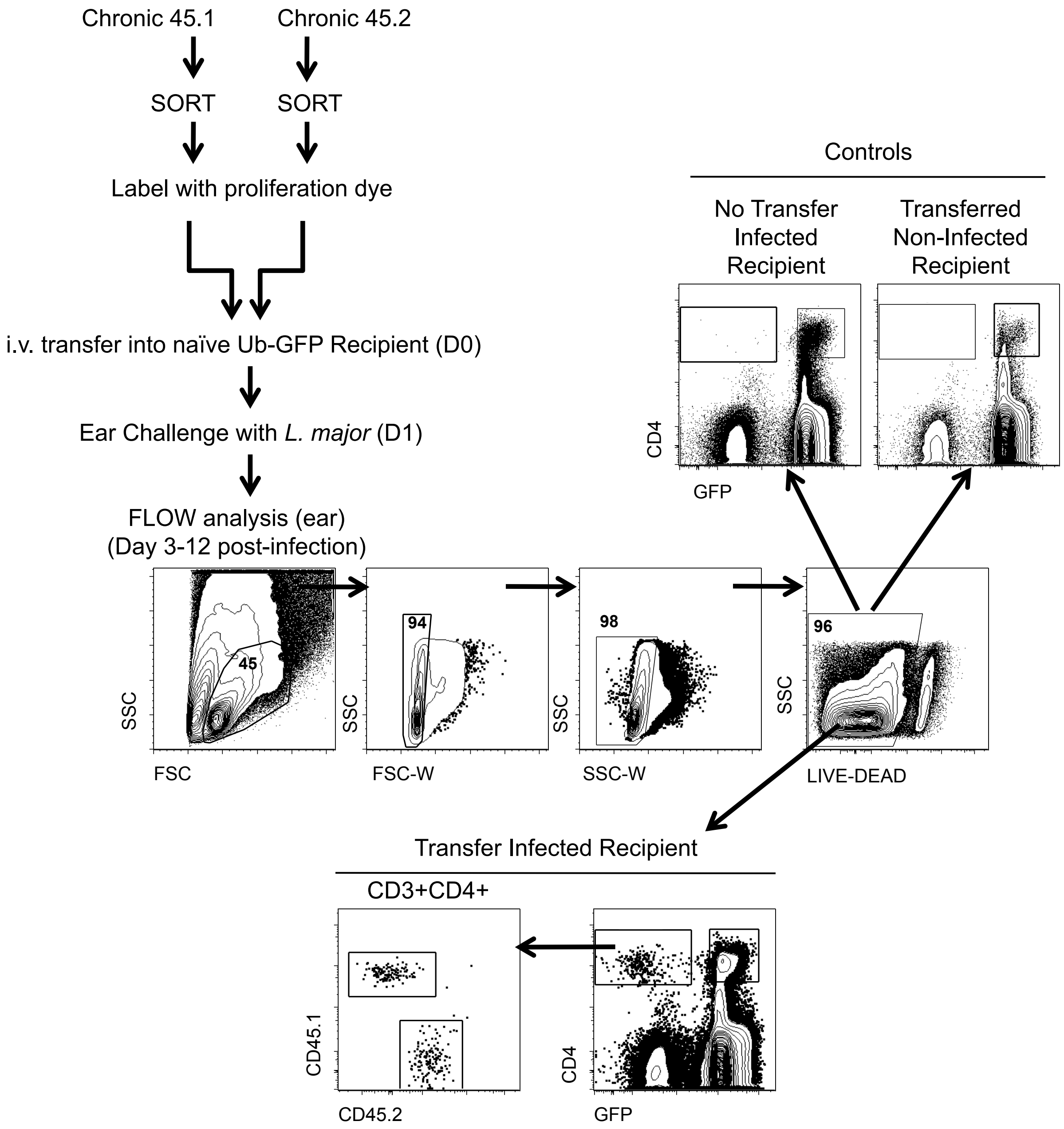

Figure S2. Representative gating strategy for detection of co-transferred, sorted cells from chronic congenic mice in naïve UB-gfp recipients. Age-matched female CD45.1 and CD45.2 mice were infected in the LHFP with *L. major*. 16-20 weeks later mice were sacrificed and cells sorted from the spleens, dLN, and ndLNs. Different sorted populations were labeled with a proliferation dye and co-transferred into naïve CD45.2 UB-gfp recipients. One day later mice were infected in the ear dermis with *L. major*. Following infection cells were analyzed by flow cytometry according to the depicted gating strategy. Inter- and intra-ear comparisons of different sorted populations generated similar results.
