# Supplementary material for: Chronic Parasitic Infection Maintains High Frequencies of Short-Lived Ly6C+CD4+ Effector T Cells That Are Required for Protection against Re-infection
Source: PLoS Pathog. 2014 Dec 4;10(12):e1004538. doi: 10.1371/journal.ppat.1004538 (PMC4256462; doi:10.1371/journal.ppat.1004538)
Supplement: Figure S3 — Phenotypic analysis of polyclonal CD3+CD4+ T cells from mice chronically with L. major . Naïve (CD44−CD62L+, black line), TCM (CD44−CD62L+, red line), or TEFF/TEM CD44+CD62L− CD4+ T cells (blue line) from chronic mice were analyzed for expression of the indicated markers. (B) Analysis of the frequency of IL-7R negative/low cells or IL-7R MFI within the indicated populations. p<0.0001, n = 5. (PDF) [file ppat.1004538.s003.pdf]

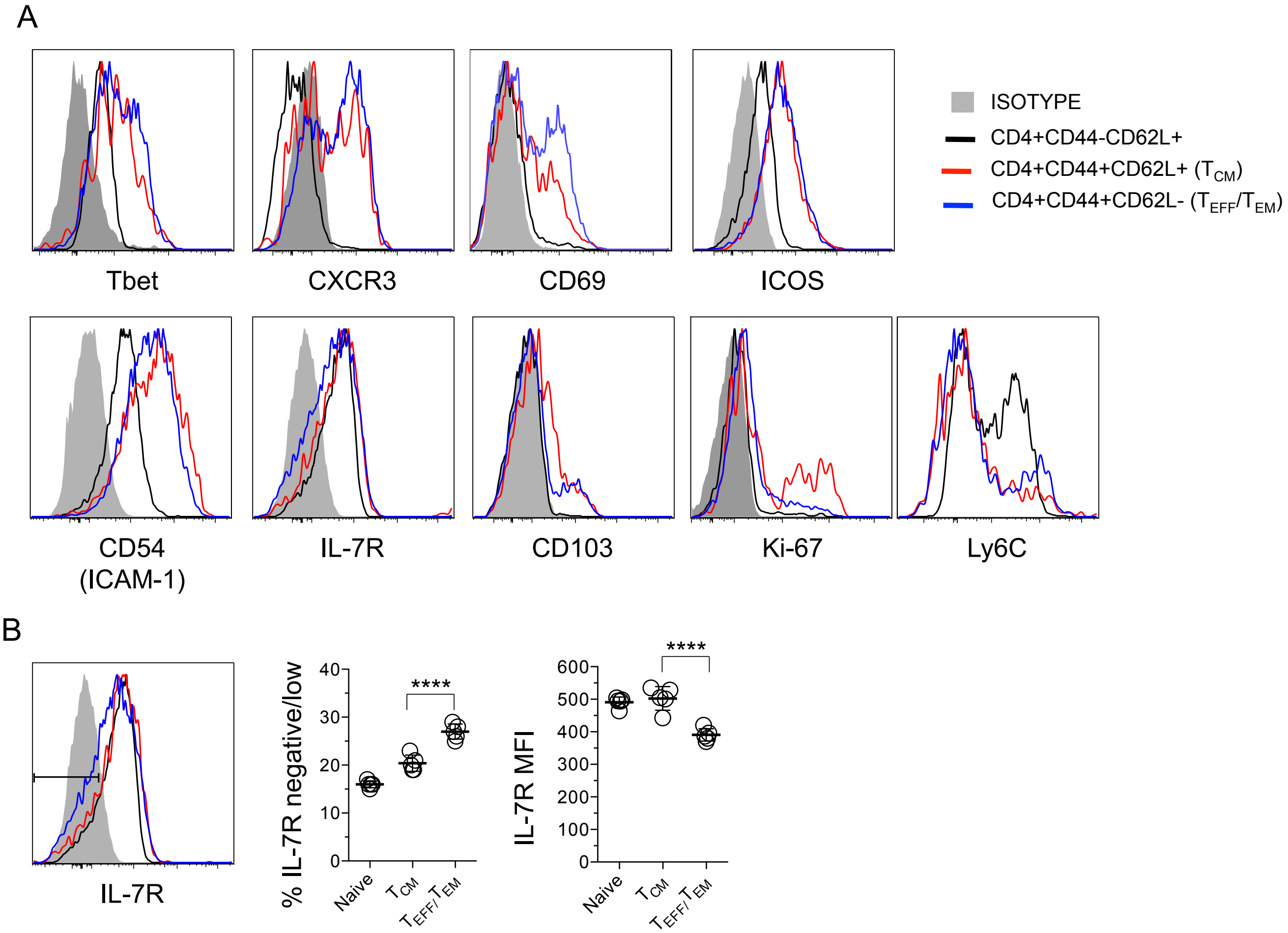

Figure S3. Phenotypic analysis of polyclonal CD3$^+$CD4$^+$ T cells from mice chronically with *L. major*. (A) Naïve (CD44$^-$CD62L$^+$, black line), T_{CM} (CD44$^-$CD62L$^+$, red line), or T_{EFF}/T_{EM} CD44$^+$CD62L$^-$ CD4$^+$ T cells (blue line) from chronic mice were analyzed for expression of the indicated markers. **(B)** Analysis of the frequency of IL-7R negative/low cells or IL-7R MFI within the indicated populations. $p<0.0001$, n=5.
