# Supplementary material for: Chronic Parasitic Infection Maintains High Frequencies of Short-Lived Ly6C+CD4+ Effector T Cells That Are Required for Protection against Re-infection
Source: PLoS Pathog. 2014 Dec 4;10(12):e1004538. doi: 10.1371/journal.ppat.1004538 (PMC4256462; doi:10.1371/journal.ppat.1004538)
Supplement: Figure S4 — Homing of CD44+CD62L− TEFF/TEM cells to the ear and proliferation of CD44−CD62L+ TCM cells in the lymph nodes does not occur in naïve recipient mice. CD4+CD44+CD62L+ (TCM) and CD4+CD44+CD62L− T cells were FACS sorted from chronic congenic mice, labeled with VIOLET proliferation dye and co-transferred into naïve UB-gfp mice (see Fig. S2). Recipient mice were not challenged (Naïve) or challenged with L. major one day post-adoptive transfer (L.m. day 1 post-A.T.). Adoptively transferred CD4+ T cells from the ear, dLN and spleen (SPL) of recipient mice were analyzed by flow cytometry on day 4 post-infection (day 4 p.i.). Representative dot-plot gated on GFP−TCRβ+CD4+ donor cells. (PDF) [file ppat.1004538.s004.pdf]

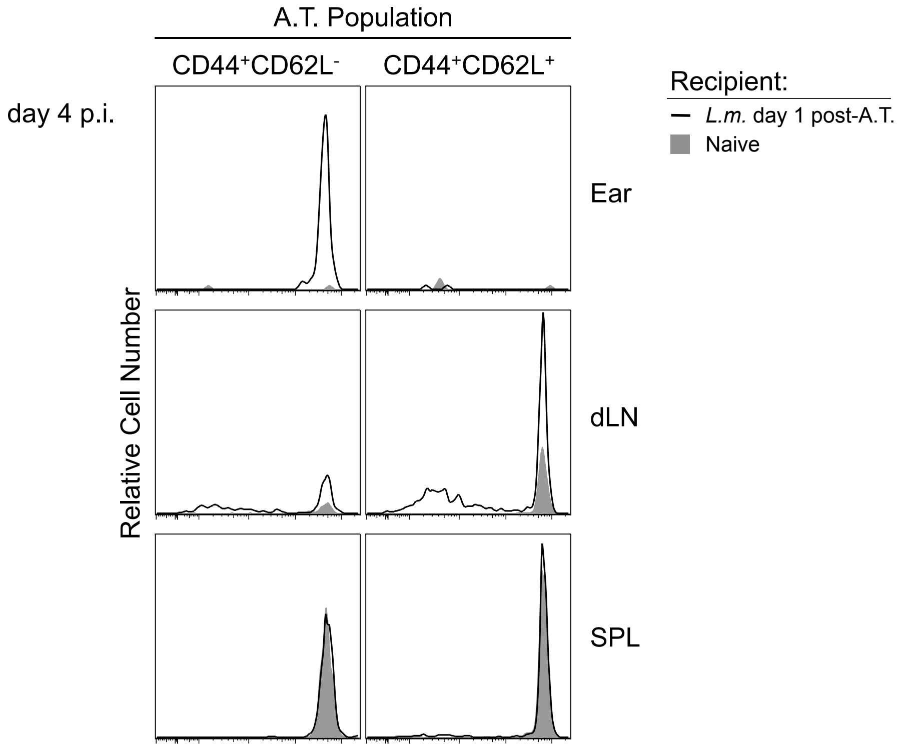

Figure S4. Homing of CD44$^+$CD62L$^-$ T$_{EFF}$/T$_{EM}$ cells to the ear and proliferation of CD44$^+$CD62L$^+$ T$_{CM}$ cells in the draining lymph nodes (dLN) does not occur in naïve recipient mice. CD4$^+$CD44$^+$CD62L$^+$ (T$_{CM}$) and CD4$^+$CD44$^+$CD62L$^-$ T cells were FACS sorted from chronic congenic mice, labeled with VIOLET proliferation dye and co-transferred into naïve UB-gfp mice (see Fig. S2). Recipient mice were not challenged (Naïve) or challenged with *L. major* one day post-adoptive transfer (*L.m.* day 1 post-A.T.). Adoptively transferred CD4$^+$ T cells from the ear, dLN and spleen (SPL) of recipient mice were analyzed by flow cytometry on day 4 post-infection (day 4 p.i.). Representative dot-plot gated on GFP$^-$TCRβ^+CD4$^+$ donor cells.
