# Supplementary material for: Chronic Parasitic Infection Maintains High Frequencies of Short-Lived Ly6C+CD4+ Effector T Cells That Are Required for Protection against Re-infection
Source: PLoS Pathog. 2014 Dec 4;10(12):e1004538. doi: 10.1371/journal.ppat.1004538 (PMC4256462; doi:10.1371/journal.ppat.1004538)
Supplement: Figure S5 — Central memory CD4+ T cells acquire the capacity to produce IFN-γ only after expansion in the dLN. (A) CD4+CD44+CD62L+ (TCM) and CD4+CD44+CD62L− T cells were FACS sorted from chronic congenic mice, labeled with VIOLET proliferation dye and co-transferred into naïve UB-gfp mice (see Fig. S2). One day post-transfer recipient mice were challenged with L. major. Adoptively transferred CD4+ T cells from the dLN of recipient mice were analyzed at the indicated time-points post-challenge by flow cytometry. Representative dot-plot gated on GFP−TCRβ+CD4+ donor cells. (B) TCM cells from chronic mice were transferred into naïve congenic recipient mice and analyzed for CD62L expression on day 12 post-infection, day 13 post-adoptive transfer. (PDF) [file ppat.1004538.s005.pdf]

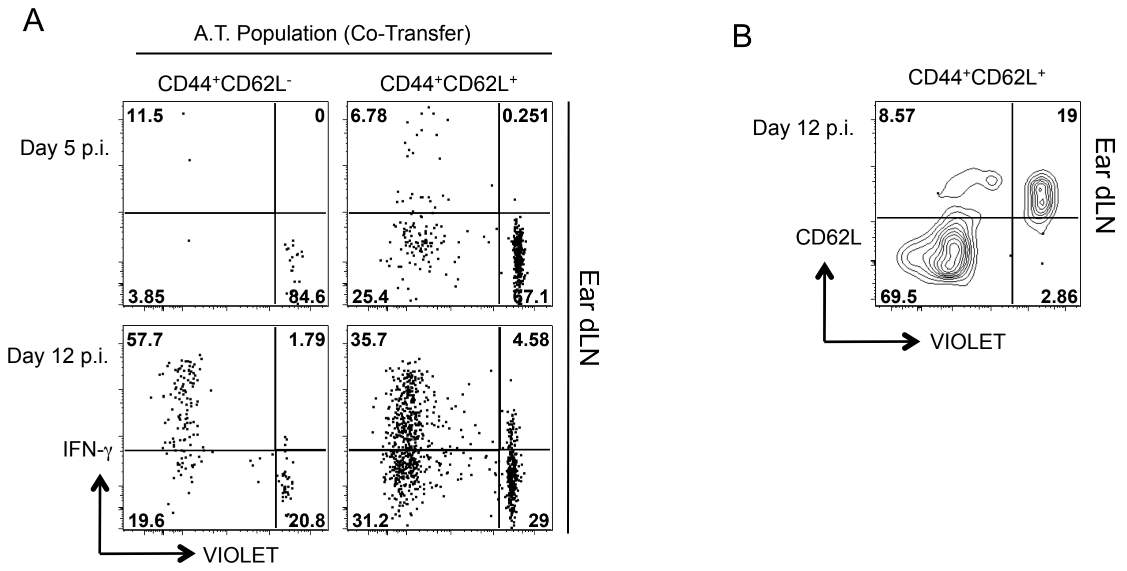

Figure S5. Central memory CD4^{+} T cells acquire the capacity to produce IFN-γ only after expansion in the dLN. (A) CD4^{+}CD44^{+}CD62L^{+} (T_{CM}) and CD4^{+}CD44^{+}CD62L^{-} T cells were FACS sorted from chronic congenic mice, labeled with VIOLET proliferation dye and co-transferred into naïve UB-gfp mice (see Fig. S2). One day post-transfer recipient mice were challenged with *L. major*. Adoptively transferred CD4^{+} T cells from the dLN of recipient mice were analyzed at the indicated time-points post-challenge by flow cytometry. Representative dot-plot gated on GFP^{-}TCRβ^{+}CD4^{+} donor cells. **(B)** T_{CM} cells from chronic mice were transferred into naïve congenic recipient mice and analyzed for CD62L expression on day 12 post-infection, day 13 post-adoptive transfer.
