# Supplementary material for: Chronic Parasitic Infection Maintains High Frequencies of Short-Lived Ly6C+CD4+ Effector T Cells That Are Required for Protection against Re-infection
Source: PLoS Pathog. 2014 Dec 4;10(12):e1004538. doi: 10.1371/journal.ppat.1004538 (PMC4256462; doi:10.1371/journal.ppat.1004538)
Supplement: Figure S6 — Phenotypic analysis of IFN-γ+ versus IFN-γ− polyclonal CD3+CD4+ T cells in the ear of chronic mice following challenge. (A and B) Uninfected ears of mice with a chronic infection in the footpad were challenged by needle inoculation with 2×10^5^ L. major. 20 hours post-challenge CD3+CD4+ T cells from the ear were analyzed by flow-cytometry employing dICS. (A) Representative histograms of IL-7R, CD27, and Ly6C expression on IFN-γ+ (blue line) versus IFN-γ− (red line) cells from the dermal site of challenge, gated as shown in Figure S1. (B) Analysis of the median fluorescent intensity of the indicated surface markers on IFN-γ+ versus IFN-γ− CD3+CD4+ T cells. ***, p<0.001, n = 4 ears. (PDF) [file ppat.1004538.s006.pdf]

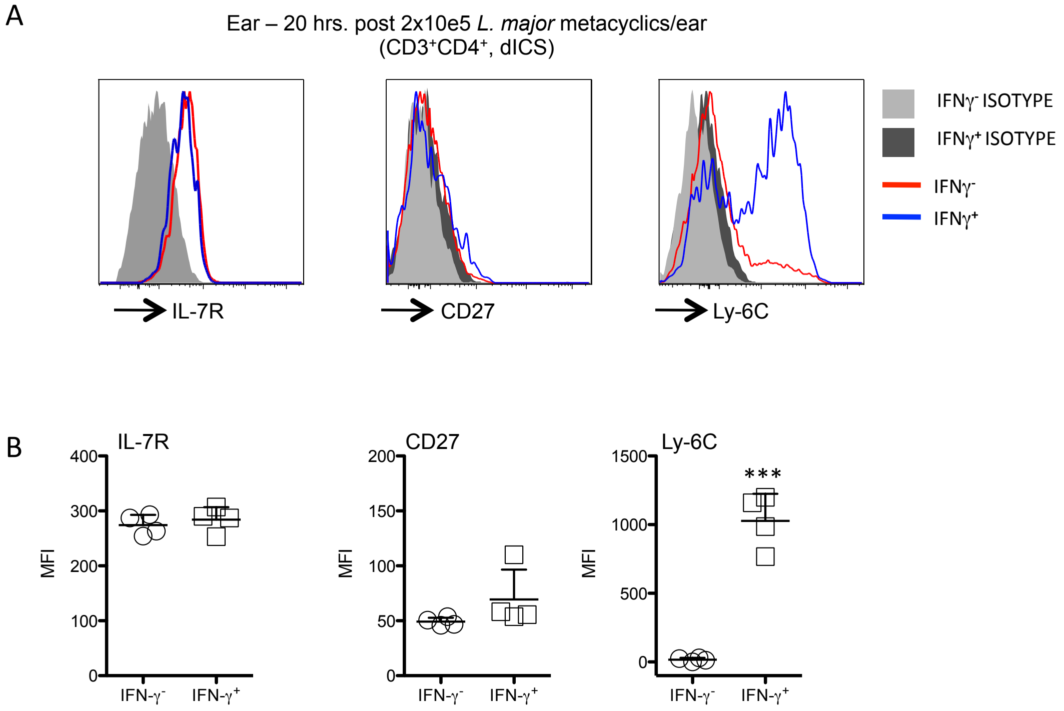

Figure S6. Phenotypic analysis of IFN-γ^{+} versus IFN-γ^{-} polyclonal CD3^{+}CD4^{+} T cells in the ear of chronic mice following challenge. (A and B) Uninfected ears of mice with a chronic infection in the footpad were challenged by needle inoculation with 2x10^{5} *L. major*. 20 hours post-challenge CD3^{+}CD4^{+} T cells from the ear were analyzed by flow-cytometry employing dICS. **(A)** Representative histograms of IL-7R, CD27, and Ly6C expression on IFN-γ^{+} (blue line) versus IFN-γ^{-} (red line) cells from the dermal site of challenge, gated as shown in Figure S1. **(B)** Analysis of the median fluorescent intensity of the indicated surface markers on IFN-γ^{+} versus IFN-γ^{-} CD3^{+}CD4^{+} T cells. ***, $p<0.001$, n=4 ears.
