# Supplementary material for: Chronic Parasitic Infection Maintains High Frequencies of Short-Lived Ly6C+CD4+ Effector T Cells That Are Required for Protection against Re-infection
Source: PLoS Pathog. 2014 Dec 4;10(12):e1004538. doi: 10.1371/journal.ppat.1004538 (PMC4256462; doi:10.1371/journal.ppat.1004538)
Supplement: Figure S7 — Phenotypic analysis of polyclonal CD3+CD4+ T cells from chronic mice. Naïve CD44−CD62L+ (black line), CD44+CD62L+Ly6C+ (red line), or CD44+CD62L−Ly6C− (blue line) T cells from chronic mice were analyzed for expression of the indicated markers. (B) Representative dot plot of Ki-67 expression versus IL-7R expression on the indicated populations in the dLN of chronically infected mice. (PDF) [file ppat.1004538.s007.pdf]

A

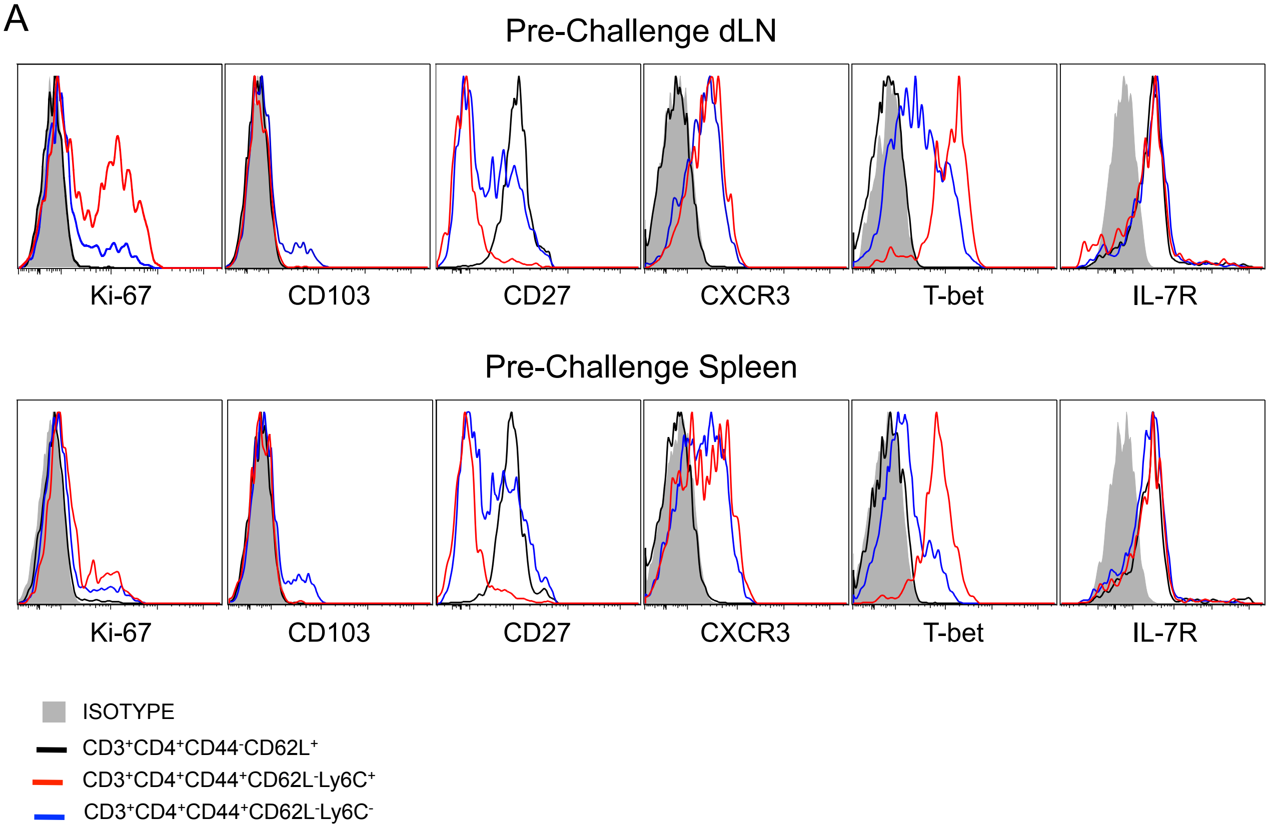

B

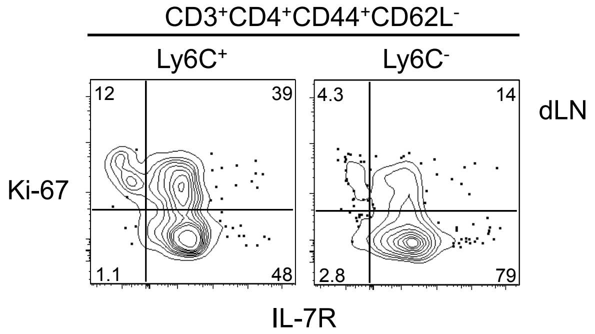

Figure S7. Phenotypic analysis of polyclonal $CD3^+CD4^+$ T cells from chronic mice. **(A)** Naïve $CD44^-CD62L^+$ (black line), $CD44^+CD62L^+Ly6C^+$ (red line), or $CD44^+CD62L^-Ly6C^-$ (blue line) T cells from chronic mice were analyzed for expression of the indicated markers. **(B)** Representative dot plot of Ki-67 expression versus IL-7R expression on the indicated populations in the dLN of chronically infected mice.
