# Supplementary material for: Chronic Parasitic Infection Maintains High Frequencies of Short-Lived Ly6C+CD4+ Effector T Cells That Are Required for Protection against Re-infection
Source: PLoS Pathog. 2014 Dec 4;10(12):e1004538. doi: 10.1371/journal.ppat.1004538 (PMC4256462; doi:10.1371/journal.ppat.1004538)
Supplement: Figure S8 — Total cell recovery of TCM, CD44+CD62L−Ly6C+, or CD44+CD62L−Ly6C− derived cells 14 days following transfer into infection matched congenic recipients. Equivalent numbers of FACS sorted cells were adoptively transferred into infection matched congenic recipients as described in Figure 8. Fourteen days post-transfer purified CD4+ T cells from the dLN and spleens of recipient mice were analyzed for the presence of transferred cells by flow cytometry. Analysis of the absolute number of total, proliferated, or non-proliferated cells per organ or per mouse (dLN+SPL) derived from the indicated transferred population. (PDF) [file ppat.1004538.s008.pdf]

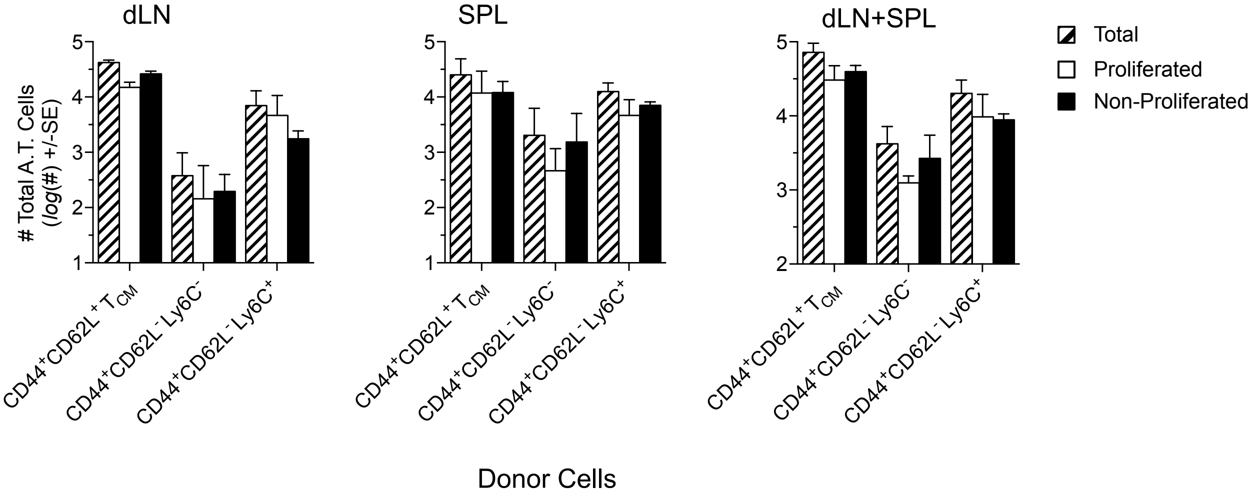

Figure S8. Total cell recovery of T_{CM}, $CD44^{+}CD62L^{-}Ly6C^{+}$, or $CD44^{+}CD62L^{-}Ly6C^{-}$ derived cells 14 days following transfer into infection matched congenic recipients. Equivalent numbers of FACS sorted cells were adoptively transferred into infection matched congenic recipients as described in Figure 8. Fourteen days post-transfer purified CD4+ T cells from the dLN and spleens of recipient mice were analyzed for the presence of transferred cells by flow cytometry. Analysis of the absolute number of total, proliferated, or non-proliferated cells per organ or per mouse (dLN+SPL) derived from the indicated transferred population.
